# Global analysis of polysome-associated mRNA in vesicular stomatitis virus infected cells

**DOI:** 10.1371/journal.ppat.1007875

**Published:** 2019-06-21

**Authors:** William J. Neidermyer, Sean P. J. Whelan

**Affiliations:** Department of Microbiology & Immunobiology, Program in Virology, Harvard Medical School, Boston, Massachusetts, United States of America; Stanford University, UNITED STATES

## Abstract

Infection of mammalian cells with vesicular stomatitis virus (VSV) results in the inhibition of cellular translation while viral translation proceeds efficiently. VSV RNA synthesis occurs entirely within the cytoplasm, where during transcription the viral polymerase produces 5 mRNAs that are structurally indistinct to cellular mRNAs with respect to their 5′ cap-structure and 3′-polyadenylate tail. Using the global approach of massively parallel sequencing of total cytoplasmic, monosome- and polysome-associated mRNA, we interrogate the impact of VSV infection of HeLa cells on translation. Analysis of sequence reads in the different fractions shows >60% of total cytoplasmic and polysome-associated reads map to the 5 viral genes by 6 hours post-infection, a time point at which robust host cell translational shut-off is observed. Consistent with an overwhelming abundance of viral mRNA in the polysome fraction, the reads mapping to cellular genes were reduced. The cellular mRNAs that remain most polysome-associated following infection had longer half-lives, were typically larger, and were more AU rich, features that are shared with the viral mRNAs. Several of those mRNAs encode proteins known to positively affect viral replication, and using chemical inhibition and siRNA depletion we confirm that the host chaperone heat shock protein 90 (hsp90) and eukaryotic translation initiation factor 3A (eIF3A)—encoded by 2 such mRNAs—support viral replication. Correspondingly, regulated in development and DNA damage 1 (Redd1) encoded by a host mRNA with reduced polysome association inhibits viral infection. These data underscore the importance of viral mRNA abundance in the shut-off of host translation in VSV infected cells and link the differential translatability of some cellular mRNAs with pro- or antiviral function.

## Author summary

Viruses co-opt the host translational machinery and frequently suppress host cell protein synthesis. Many positive-strand RNA viruses manipulate initiation factors while bypassing their need for viral protein production using internal ribosome entry sites. Negative-strand RNA viruses and DNA viruses produce mRNAs that contain host-like 5′ cap-structures and 3′ polyadenylate tails. Those similarities necessitate a different mechanism for controlling viral versus host protein synthesis. We infected cells with vesicular stomatitis virus and sequenced polysome-associated mRNAs at 2 and 6 hours post-infection providing 2 snapshots of how infection alters translation. We present evidence that the 5 viral mRNAs outcompete cellular mRNAs for ribosomes and demonstrate that individual host mRNAs vary in the extent to which their polysome association is altered by infection. Host mRNAs that are more abundant, have longer half-lives, greater than average length, and a similar AU content to the viral mRNAs were more likely to be enriched among polysome-associated cellular mRNAs. Several of the enriched mRNAs encode proteins that promote viral replication, whereas mRNAs that exhibit the largest decrease in polysome association include those that encode antiviral functions.

## Introduction

Infection of mammalian cells by vesicular stomatitis virus (VSV) results in a profound shut-off of host cell gene expression. This host cell shut-off occurs at the level of mRNA transcription through inhibition of RNA polymerase II by the viral-encoded matrix protein (M) [1–3]. The M protein also forms a complex with ribonucleic acid export 1 (Rae1) and nucleoporin 98 (Nup98) [4] thus suppressing host cell mRNP export from the nucleus, including that of mature cellular mRNAs [5–8]. VSV infection also inhibits protein synthesis by manipulation of the host-cell translation machinery, particularly at the level of translation initiation [9, 10]. Eukaryotic initiation factor 4E (eIF4E)—the rate limiting factor for translation initiation—recognizes the 7^m^GpppN mRNA cap structure as part of the eIF4F complex, and in concert with other translation initiation factors facilitates the recruitment of the small 40S ribosomal subunit to the mRNA prior to scanning to the initiating methionine where the 60S subunit joins [11, 12]. VSV infection results in the rapid dephosphorylation of eIF4E itself, for which the functional consequences are unclear, and of its binding protein (eIF4E-BP1) leading to eIF4E sequestration and the suppression of translation initiation [9, 10].

Viral gene expression evades the shut-off mechanisms employed to suppress host gene expression. As VSV RNA synthesis occurs entirely within the cytoplasm, viral RNA synthesis is not subject to the inhibitory effects of M on RNA polymerase II and mRNA export from the nucleus. The VSV RNA synthesis machinery comprises a ribonucleoprotein complex of the negative-sense genomic RNA completely encased by a nucleocapsid protein (N) sheath and associated with the viral polymerase complex [13]. The viral transcriptase copies the N-RNA template into 5 monocistronic mRNAs that are structurally indistinct to those of the host-cell with respect to their 5′ cap and 3′ polyadenylate tail [14–20]. The enzymes necessary for mRNA synthesis, namely an RNA dependent RNA polymerase (RdRp) and a set of capping enzymes, reside within the viral large protein (L) [17, 19, 21–27]. VSV L protein cannot engage the N-RNA template directly, but instead depends on the viral phoshoprotein (P) to facilitate the interaction [28–33]. Messenger RNA polyadenylation is also catalyzed by L through reiterative transcription by the RdRp of a U7 tract that resides at the end of each gene [34–38]. This program of viral transcription results in the cytoplasmic synthesis of 5 mRNAs that depend upon the host machinery for their translation, and must therefore avoid the shut-down mechanisms that effectively suppress host mRNA translation.

Metabolic labeling studies demonstrate that 4 hours post VSV infection of baby hamster kidney cells in culture, total translation is suppressed to about 65% the level of uninfected controls [39]. Extraction of mRNA from infected cells coupled with its *in vitro* translation confirmed that the cellular mRNAs remain intact and are competent for translation [39]. The VSV mRNAs are present in an approximately 2–3 fold excess of the total cellular mRNA, leading to the model that competition between viral and cellular mRNAs for ribosomes results in the dominance of viral translation [39, 40]. Polysome analysis also demonstrates that the cellular mRNAs are associated with significantly fewer ribosomes in infected cells [39]. For example, infection results in the movement of actin mRNA from polysomes containing 12 or more ribosomes to those containing 5 [39]. This movement reflects the competition between viral and cellular mRNA for ribosomes and the limited pool of eIF4E.

The competition model predicts that the kinetics of viral mRNA synthesis and the levels of viral mRNA should correlate closely with host shut-off. Tests of this prediction yielded conflicting results. The kinetics of host shut-off and viral mRNA accumulation correlate well for many strains of VSV, consistent with the competition model [39, 40]. Inhibition of host protein synthesis is, however, largely unaffected following coinfection of cells with increasing quantities of defective interfering (DI) particles that suppress viral mRNA levels up to 14-fold [41]. A similar result was obtained for a VSV mutant that is restricted for genome replication at 39°C, and yields only 30% of the wild type levels of viral mRNA [41]. Collectively, these studies suggest additional mechanisms may contribute to the shut-off of host cell protein synthesis.

Specific features of the viral mRNAs that contribute to their efficient translation have not been defined. The 5′ untranslated regions of VSV mRNAs are short, being 10–15 nucleotides for the viral N, P and L mRNAs that encode the proteins required for RNA replication [42, 43]. How such short 5′ UTRs serve as effective initiators of translation is unclear. Evidence for differential translation of viral mRNA comes from small interfering RNA suppression of eIF4E, which inhibits host gene expression but has no impact on viral gene expression [44]. Viral translation is also hypersensitive to the loss of ribosomal protein L40, suggesting different mRNA features facilitate translation of viral versus host mRNA [45]. Flanking cellular or reporter genes by the conserved viral 10-nt gene-start and 13-nt gene-end sequences, and inserting them into the viral genome is sufficient to mediate their efficient translation [46]. By contrast, expression of the same genes following transfection of plasmid DNA into cells and subsequent VSV infection does not offer this translational advantage [46]. Thus, transcription of the mRNAs from the viral genome appears to contribute to their efficient translation.

In the present study, we interrogate global mRNA translation in VSV infected cells using RNAseq analysis of the cytoplasmic mRNA transcriptome, and parallel sequencing of polysome-associated mRNAs. We obtain support for the model that an overabundance of viral mRNA contributes to host shut-off by leading to a re-distribution of cellular ribosomes onto viral mRNA. By combining this RNAseq analysis with examining the distribution of specific viral and cellular mRNAs within polysomes, we also demonstrate that mRNAs shift to smaller polysomes. Analysis of cellular mRNAs less-sensitive to this global shut-down of translation identifies several host proteins that promote viral replication. Similar analyses revealed the abundance of viral mRNA contributes to the host-cell shut-off for other viruses including coronaviruses, influenza and vaccinia [47–49].

## Results

### Viral mRNA comprises 60% of the cytoplasmic mRNA at 6 hours post-infection

**Fig 1:**
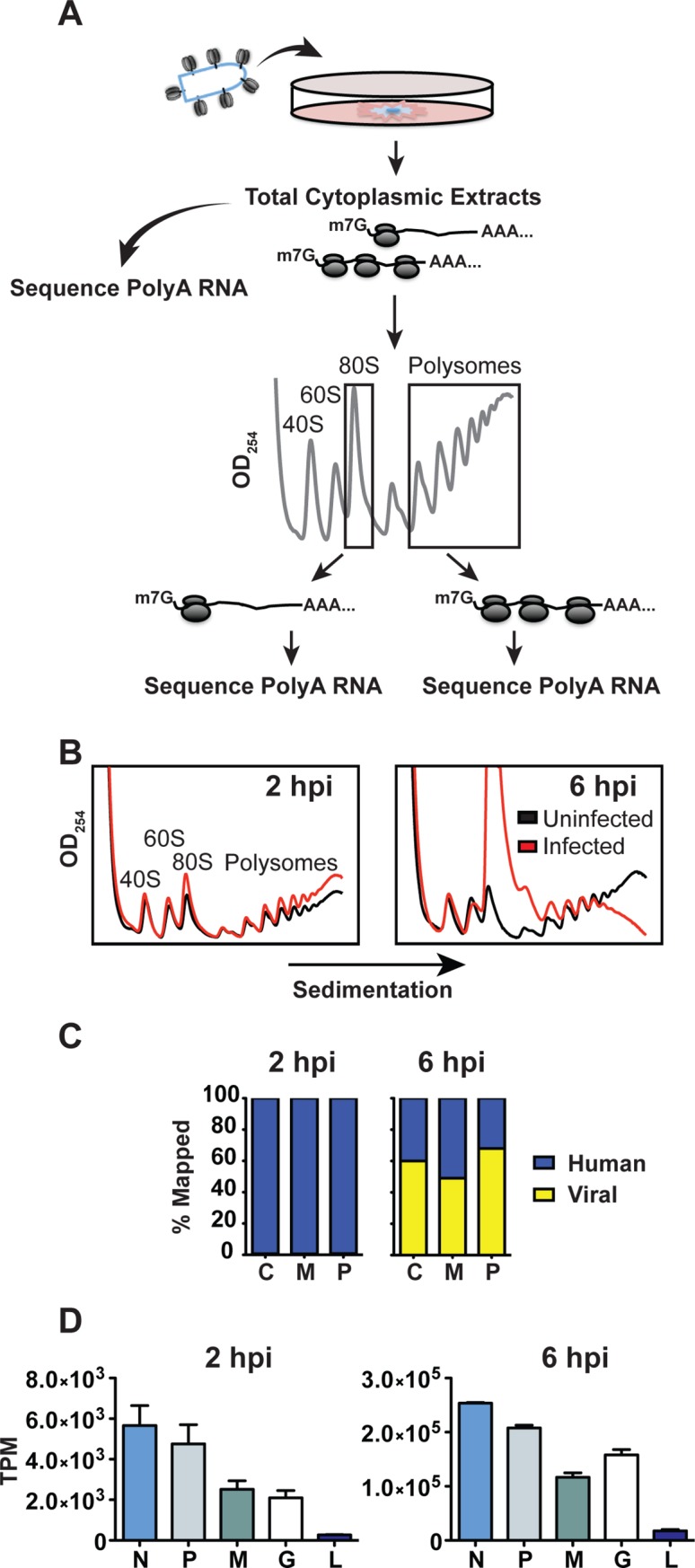
Viral mRNA comprises 60% of the cytoplasmic mRNA at 6 hours post-infection. (A) Schematic of experimental design. HeLa cells were infected with VSV at a MOI of 10 and cytoplasmic extracts were prepared at 2 and 6 hpi for mRNA isolation and polysome profiling. Messenger RNA was isolated from fractions corresponding to 80S monosomes, or polysomes containing 3 or more ribosomes, and used for deep sequencing. (B) Polysome analysis of uninfected (black) or VSV (red) infected HeLa cells. Cytoplasmic extracts were sedimented through a 10–50% sucrose gradient and 0.5 ml fractions were collected while continuously monitoring absorbance at λ = 254nm. (C) Distribution of fragments mapping to the concatenated hg38 (human) and VSV genomes for cytoplasmic, monosome, and polysome samples at 2 and 6 hpi. Trimming and mapping was performed in CLC Genomics Workbench. (D) Distribution of reads among the 5 viral genes at 2 and 6 hpi. Expression level is presented as Transcripts per Kilobase Million (TPM) to normalize for gene length and library size, error bars denote standard deviation from two biological replicates.

To interrogate the impact of VSV infection on global translation we isolated total cytoplasmic, monosome- and polysome-associated mRNA from HeLa cells at 2 and 6 hpi and compared the relative sequence reads obtained by deep-sequencing (Fig 1A). Statistical analysis of sequencing reads between biological replicates from each fraction yields a Pearson correlation of >0.97 for cytoplasmic, monosome- and polysome-associated mRNA pools validating reproducibility between the replicates.

As visible in the polysome profiles (Fig 1B), VSV infection results in a small but reproducible increase in the pool of monosomes and large polysomes at 2 hpi, and a collapse of large polysomes and an increase in monosomes by 6 hpi. Mapping of the sequence reads to the viral and host genome highlights that by 6 hpi >60% of the total reads in the cytoplasmic and polysome fractions are viral (Fig 1C). This increase from the <1% observed at 2 hpi (Fig 1C) emphasizes the impact of the exponential phase of viral RNA replication and secondary transcription of the viral genome on mRNA production. The viral sequence reads map to all 5 genes, with clear dips in coverage at gene-junctions (S1 Fig). Consistent with the order of transcription of the viral genome and the localized transcriptional attenuation at gene-junctions [50–53], the relative reads that map to each viral gene generally diminish with distance from the single 3′ promoter (Fig 1D and S1 Fig).

Analysis of the sequencing reads that map to cellular genes supports that like the viral mRNAs, the level of reads in the polysome fraction mirrors that in the total cytoplasmic fraction at 2 and 6 hpi (Fig 1C). This result demonstrates that the majority of mRNAs are polysome-associated in proportion to their abundance. The abundance of the 5 viral mRNAs at 6 hpi supports the model that viral mRNAs outcompete cellular mRNAs for ribosomes [39]. We note that viral mRNAs are, however, underrepresented (49%) and cellular mRNAs overrepresented (51%) in the monosome fraction at 6 hpi, compared to their cytoplasmic abundance (Fig 1C). This finding is consistent with a differential effect on viral versus host mRNA translation.

### The relative abundance of individual cellular mRNAs in the cytoplasm and on polysomes decreases between 2 and 6 hpi

**Fig 2:**
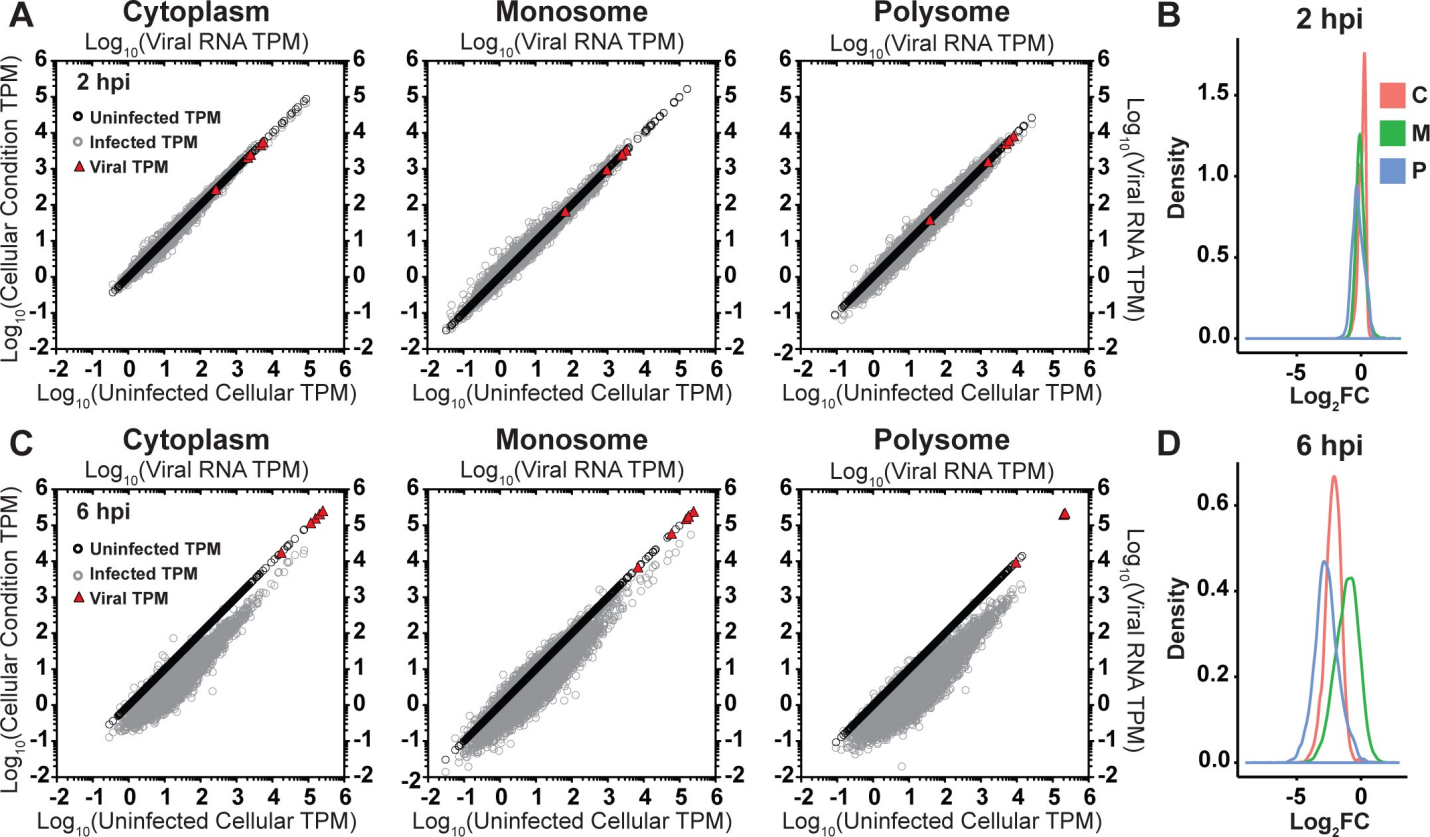
The relative abundance of individual cellular mRNAs in the cytoplasm and on polysomes decreases between 2 and 6 hpi. (A) Scatter plots of Transcripts per Kilobase Million (TPM) for exonic regions at 2 hpi. The TPM in uninfected cells for a given mRNA is graphed on the abscissa, and the TPM for a given cellular mRNA in either uninfected (black circles), or VSV infected cells (gray circles) is graphed on the ordinate. The viral mRNAs are indicated by the red triangles. (B) Density plots of the log_2_ fold change in TPM for cellular mRNAs between uninfected or VSV infected cells at 2 hpi. C, M, and P denote “cytoplasm”, “monosome”, and “polysome”, respectively. (C) Scatter plots of TPMs for individual mRNAs at 6 hpi, presented as in A. (D) Density plots of the log_2_ fold change in TPM at 6 hpi, presented as in B.

To determine how VSV infection affects the distribution of the population of mRNAs between total cytoplasmic, monosome or polysome fractions, we plotted the transcript per million (TPM) for each individual mRNA mapped to the human and viral genome in all 3 fractions (Fig 2). At 2 hpi, reads that map to the viral genes in each fraction are similar in abundance to those reads that map to highly expressed cellular genes (Fig 2A). The reads that map to any given cellular gene alter within a relatively narrow range, with few genes showing a greater than 2-fold change in the relative number of sequence reads (Fig 2B). For the population of mRNAs, the relative reads obtained from the polysome fraction mirrored the relative reads in the total cytoplasmic fraction, consistent with the abundance of an mRNA being a determinant of its translatability.

By 6 hpi, reads that map to each of the 5 viral genes—with the exception of L—exceed the reads that map to any individual cellular gene (Fig 2C, red triangles). This is concurrent with a decrease in reads that map to the majority of cellular genes in cytoplasmic, monosome and polysome-associated fractions (Fig 2C). There were, however, some distinctions between the monosome and polysome fractions. For the majority of the population of cellular mRNAs, reads were most reduced in the polysome fraction compared to the total cytoplasmic fraction (Fig 2D). A smaller reduction in reads was observed in the monosome fraction, and some cellular mRNAs even showed an increase in reads compared to the total cytoplasmic fraction (Fig 2C). This may reflect differences in the movement of cellular mRNAs from large polysomes to monosomes or out of the pool of translating ribosomes.

### Polysome association remains directly proportional to cytoplasmic abundance for cellular mRNAs at 6 hpi

**Fig 3:**
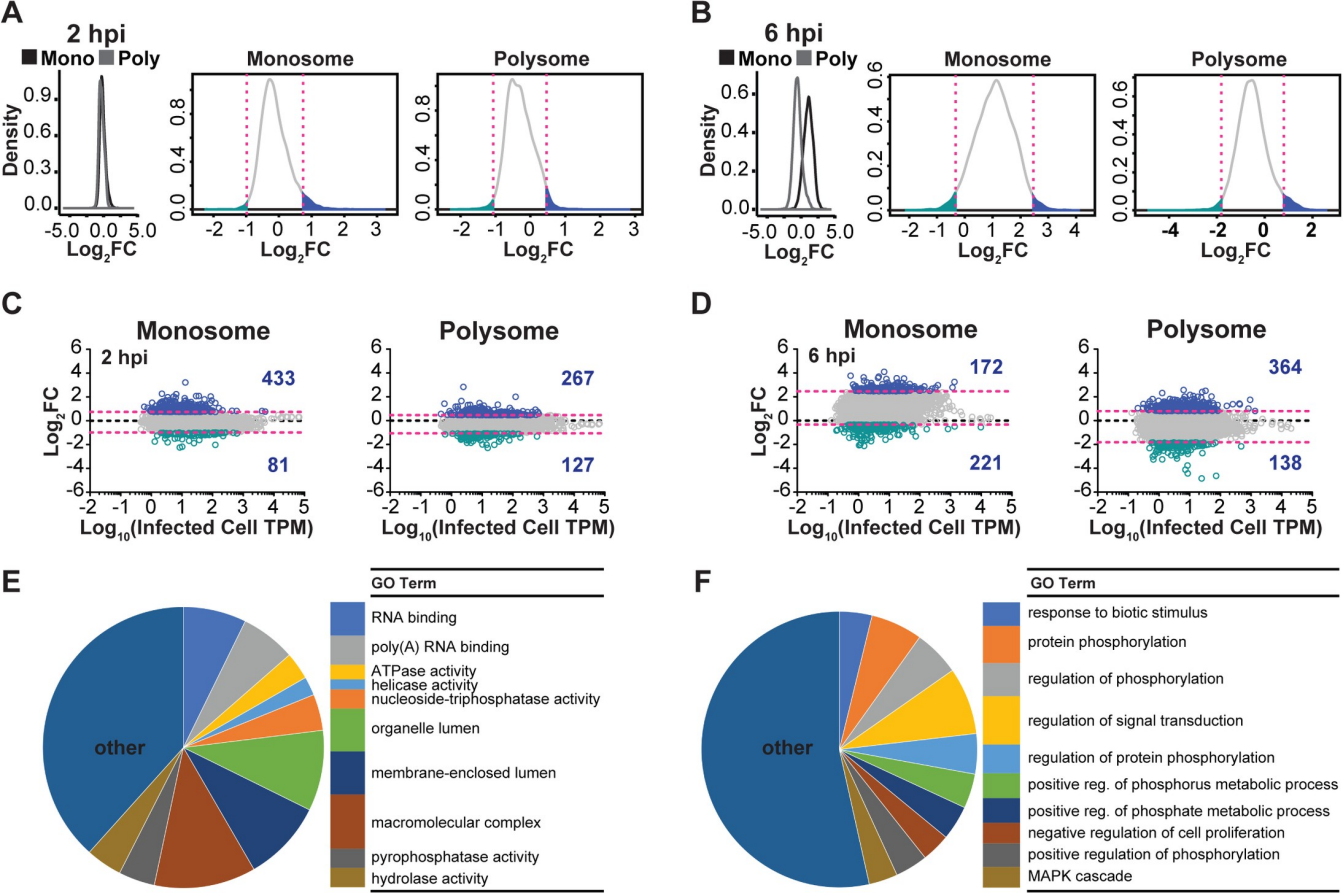
Changes in monosome and polysome association of cellular mRNAs following VSV infection. (A) Density plots show the log_2_ fold change for any given mRNA in both monosome and polysome fractions at 2 hpi. The region of the density within the 95% confidence interval of the mean is shaded gray. Magenta lines denote ± 2 standard deviations of the mean. Regions with increased association relative to the mean are shaded blue, and regions with decreased association are shaded green. (B) Analysis at 6 hpi, presented as in A. Downstream analyses were performed on genes in the green or blue regions. (C) Plots of log_2_ fold changes in association at 2 hpi plotted against cytoplasmic abundance in infected cells. Magenta lines denote ± 2 standard deviations of the mean log_2_ fold change. Genes outside the 95% confidence interval are denoted by blue (increased) or green (decreased) dots. (D) The log_2_ fold changes in association at 6 hpi as presented in C. (E) Gene ontology analysis for mRNAs with increased polysome association, analysis was performed using GOseq in R. The pie charts shown represent the distribution of mRNA among the 20 most significant GO Terms. (F) Gene ontology analysis for mRNAs with decreased polysome association as determined using GOseq in R.

We next mined our sequence data for evidence of differential translation of cellular mRNAs following VSV infection. For this analysis we divided the polysome TPM by the total cytoplasmic TPM as an indicator of the efficiency with which any given mRNA is translated. We also performed a similar analysis for the monosome pool. We are cognizant of the fact that such ratios ignore the movement of any given mRNA from larger to smaller polysomes, and will likely represent an underestimate of the extent of any translational regulation. To identify the subset of the population of cellular mRNAs with the highest probability for translational regulation in infected cells, we plotted the fold change in TPM at 2 and 6 hpi (Fig 3A–3D). At 2 hpi the monosome or polysome-associated reads changed within a narrow range for the majority of cellular genes (Fig 3A). The marked shut-off of host protein synthesis observed by metabolic labeling suggests that at 6 hpi the association of cellular mRNA with polyribosomes would alter significantly at the population level. Although we observe a global reduction in polysome-associated reads for the bulk of the population of cellular mRNAs the reduction is less than 2–3 fold. Accompanying this global reduction in polysome-associated reads, we also observe an increase in monosome-associated reads with more than half the mRNAs within the population exhibiting a >2-fold increase (Fig 3B and 3D). From the above ratios we selected the subset of cellular mRNAs that exhibit the largest changes in relative polysome-associated reads at 6 hpi to determine whether those mRNAs shared any common features. For this purpose, we selected those mRNAs that change >2 standard deviations of the mean and thus exceed the 95% confidence interval. This analysis identified 364 cellular mRNAs as candidates for translational upregulation and 138 cellular mRNAs as candidates for translational downregulation following VSV infection (Fig 3B and 3D). Consistent with monosome and polysome-associated reads at 2 hpi changing within a narrow range, only 4 genes with increased and 20 with decreased polysome association, overlap between 2 and 6 hpi (Fig 3C and 3D). Within the monosome fraction 8 genes with increased and 6 with decreased monosome association overlap from 2 to 6 hpi.

### Cellular mRNAs that exhibit evidence of positive translational regulation are AU-rich and longer

We next determined whether shared functional or sequence elements are present within the specific subsets of 364 mRNAs with increased polysome association, or the 138 mRNAs with decreased polysome association (Fig 3B and 3D, blue and green dots). For the 364 genes with significantly increased polysome-associated reads, gene ontology analysis identifies functions in RNA binding, helicase and NTPase activities, among others (Fig 3E and S3 Dataset). The 138 genes with decreased polysome-associated reads are associated with cellular responses to stimuli and signaling activities (Fig 3F and S3 Dataset). This gene ontology analysis reveals that the up and down regulated transcripts comprise distinct functional groups.

**Fig 4:**
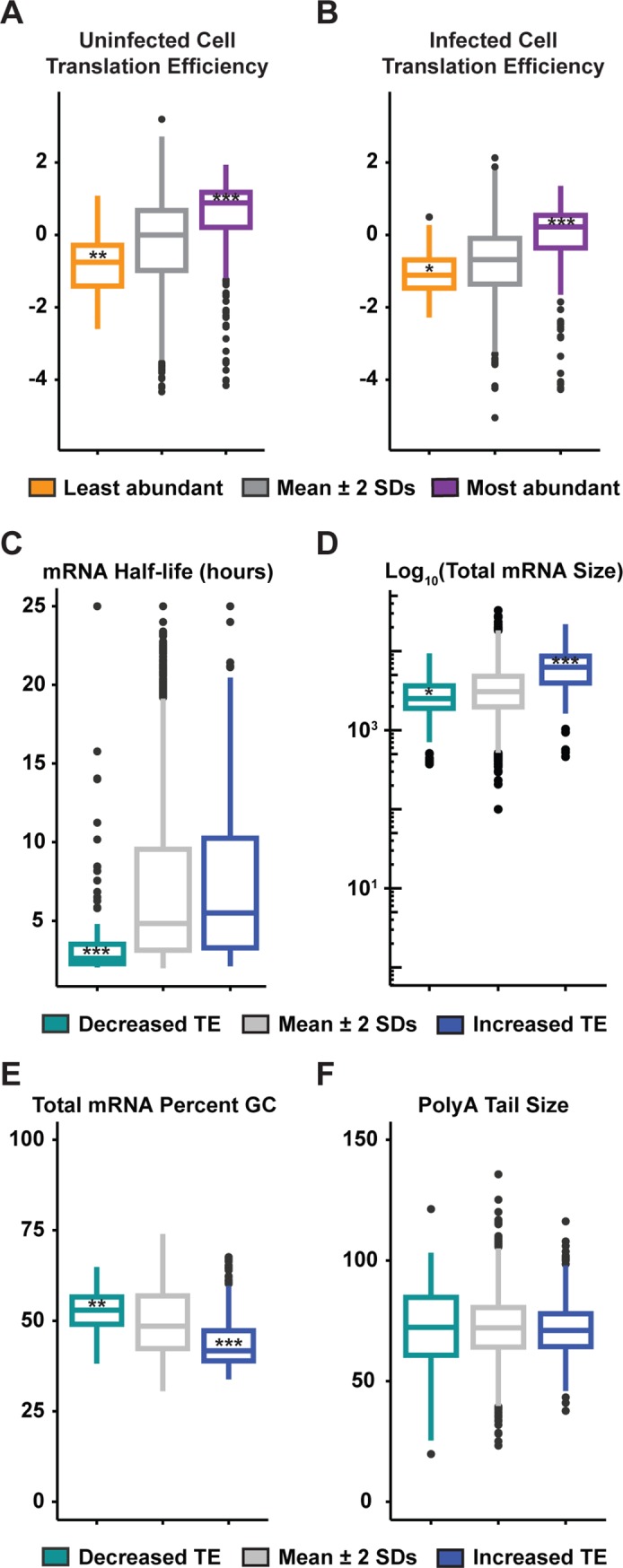
Polysome-associated cellular mRNAs are longer and more AU-rich. (A) Analysis of cellular mRNAs with high cytoplasmic abundance (purple) or low cytoplasmic abundance (orange) as compared to mRNAs with cytoplasmic abundance within 2 standard deviations of the mean abundance (gray) in uninfected cells. Cytoplasmic abundance by TPM is from the data set published with this paper. ***p< 2.2 x 10^−16^; **p< 5.0 x 10^−5^; *p< 0.05; all others p> 0.05 as determined by the Wilcoxon rank sum test compared to mRNAs with relative abundance levels within the 95% confidence interval of the mean. Hinges correspond to the 25th-75th percentiles, and whiskers denote 1.5 times the inter-quartile range. (B) Analysis as in A for cytoplasmic abundance in infected cells. (C-F) mRNA characteristics for mRNAs with increased polysome association (blue) or decreased polysome association (green) at 6 hpi, as defined in Fig 3. Data for RNA half-life and poly(A) tail length were from [54, 55]. Analysis was performed as in A.

At 6 hpi the cytoplasmic abundance of cellular mRNAs correlates with their polysome association at 6 hpi (Fig 4A and 4B), consistent with mRNA abundance being a determinant of translatability. As described above, we use as an indicator of translation efficiency (TE) of an mRNA the ratio of polysome to total cytoplasmic associated reads. To determine whether there are shared features between the 364 mRNAs with evidence of enhanced polysome association or the 138 with reduced polysome association, we extracted mRNA sequences and annotations from the UCSC Genome Browser. Assisted by published datasets we examined whether the half-life, size, GC content or poly(A) tail length correlate with increased or decreased polysome association (Fig 4C*–*4F and S2 Fig) [54, 55]. Cellular mRNAs with increased polysome-associated reads tended to have a longer half-life, were typically larger, and were more AU-rich (Fig 4C–4E and S2 Fig). Correspondingly, those with decreased polysome-associated reads tended to have shorter half-lives, higher GC content, and were typically smaller. The correlation between higher AU content and increased polysome-associated reads was most evident for the coding region and 3′ UTR (S2 Fig). The effect of length was predominantly a determinant of the ORF and not the 5′ or 3′ UTR (S2 Fig). There was no correlation between poly(A) tail length and polysome-associated reads at 6 hpi (Fig 4F). This analysis highlights that the cellular mRNAs that exceed the 95% confidence interval for increased polysome-associated reads in response to VSV infection are most similar to the viral mRNAs in that they are typically longer and more AU rich.

### Extent of polysome association of some cellular mRNAs correlates with pro- or antiviral protein function

**Fig 5:**
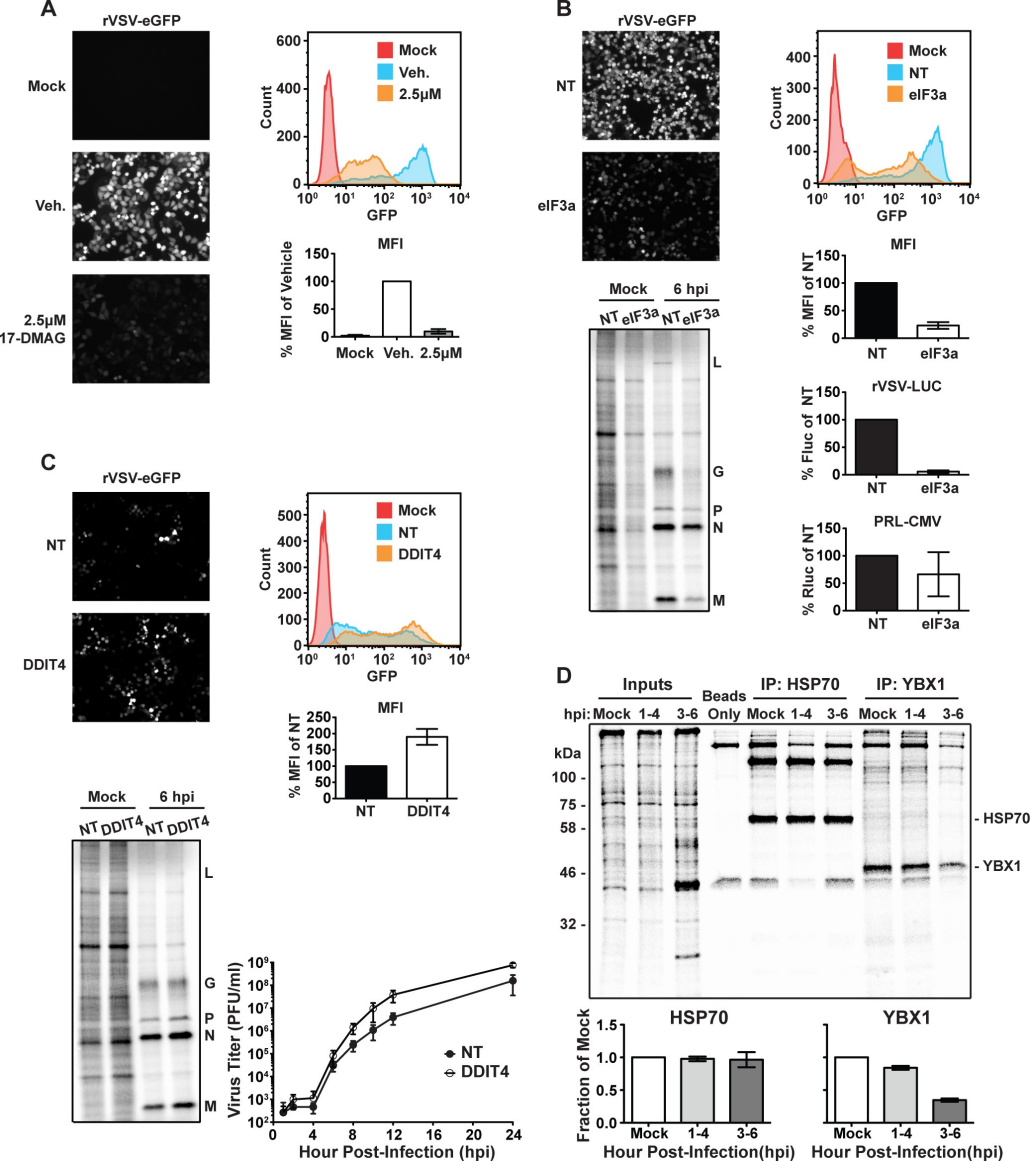
Effect of suppressing specific host gene function on viral gene expression. (A) GFP expression of a rVSV-eGFP reporter virus in uninfected, vehicle-treated, or 2.5 μM 17-DMAG treated HeLa cells. A representative histogram of GFP intensity is shown to the right, and the mean fluorescence intensity (MFI) of live cells is shown below. MFI is normalized to vehicle, and error bars denote the standard deviation from the mean of 3 independent replicates. (B) VSV gene expression in eIF3a depleted HeLa cells. A representative histogram of fluorescence intensity is shown to the right and the MFI below. Error bars denote standard deviation from the mean from three independent replicates. Luciferase expression driven by rVSV-LUC (Firefly Luciferase) or a cellular reporter driving expression of Renilla Luciferase (pRL-CMV) in eIF3a depleted cells. Luciferase expression is presented as the percent of a non-targeting siRNA control, and error bars denote the standard deviation from 3 independent replicates. Metabolic labeling of eIF3a depleted HeLa cells infected with wild-type VSV. The position of viral proteins is noted to the right. Presented is a representative gel from two independent replicates. (C) VSV gene expression and replication in DDIT4 depleted cells. A representative histogram of eGFP expression is shown along with the MFI of cells normalized to a non-targeting siRNA control. Error bars denote the standard deviation from the mean of three independent replicates. For metabolic labeling a representative gel of two independent replicates is presented. Kinetics of viral replication were measured by titration of yields at various times post infection of siRNA treated HeLa cells. Error bars denote the standard deviation from the mean of 3 independent replicates. (D) Immunoprecipitation of cellular proteins synthesized post-infection with VSV_WT_. Shown is a representative gel from two independent replicates. A quantitative analysis of the HSP70 and YBX1 bands is shown in the bottom two panels, error bars denote the standard deviation from two independent replicates.

We next examined whether cellular mRNAs that exhibit increased polysome association encode proteins that are pro- or antiviral. Among the 364 cellular mRNAs with increased polysome association several encode known proviral factors including the heat shock proteins (HSP) 90, 70, and 60. Previous work demonstrated that inhibition of HSP90 inhibits viral replication, and linked inhibition of those chaperones to defects in L protein folding [56–58]. We independently verified the proviral function of HSP90 using the inhibitor 17-[2-(Dimethylamino)ethyl]amino-17-desmethoxygeldanamycin (17-DMAG) [59, 60]. Infection of HeLa cells with VSV that expresses eGFP as a marker of infection demonstrates that 17-DMAG has no effect on the fraction of cells infected, but slows the rate of eGFP expression (Fig 5A and S3 Fig). This was not simply due to defects in eGFP folding, as metabolic labeling of viral RNA substantiates the defect in gene expression (S3 Fig).

We also found that polysome association of the mRNA encoding eukaryotic initiation factor 3 subunit A (eIF3a), increases after infection. To test whether this reflects a specific proviral function of eIF3a, we used siRNA depletion to reduce eIF3a and measured viral gene expression using reporter viruses expressing eGFP or luciferase. Both reporter viruses displayed a sensitivity to the loss of eIF3a (Figs 5B and S3). As expected, depletion of eIF3a also reduced cellular translation in uninfected cells, but that reduction was modest as evidenced by expression of a CMV promoter driven renilla luciferase reporter (Fig 5B). Translation of the CMV driven reporter, however, reflects the accumulated steady-state levels of luciferase mRNA. We therefore measured the effect of eIF3a depletion on viral vs host translation by metabolic incorporation of [^35^S]-methionine in infected and uninfected cells (Fig 5B). Following eIF3a depletion we observed a 55% reduction in viral M protein synthesis over a 30 minute time period, which is similar to the 56% reduction in host protein synthesis measured in mock infected cells. This analysis supports a proviral role for 2 cellular mRNAs that encode proteins with important house-keeping functions that remain polysome-associated following VSV infection.

Among the 138 cellular mRNAs that exhibit reduced polysome association following infection was DNA-damage inducible transcript 4 (DDIT4) which encodes regulated in development and DNA-damage response 1 (Redd1) [61–63]. Existing studies demonstrate that DDIT4/Redd1 restricts the replication of negative-strand RNA viruses, including VSV. Depletion of DDIT4/Redd1 by siRNA increased viral gene expression as evidenced from infection of cells with VSV-eGFP, and by metabolic labeling of viral protein synthesis (Fig 5C, S3 Fig). Consistent with the enhancement of viral gene expression following DDIT4 depletion, we obtained an approximately 10-fold increase in viral titers (Fig 5C). Depletion of DDIT4/Redd1 also increases cellular protein synthesis likely reflecting its role as a negative regulator of mTOR (Fig 5C and S3 Fig).

The above analysis supports that the polysome association of some host mRNAs following VSV infection correlates with their pro- or antiviral functions, but does not directly demonstrate that the level of polysome association is associated with a change in synthesis of the corresponding protein. To independently examine whether changes in polysome association of host mRNAs affect synthesis of the corresponding protein we selected the heat shock protein (HSP70) and Y-box binding protein 1 (YBX1) as representative mRNAs with increased and decreased polysome association, respectively. We selected those mRNAs based on their high-levels of expression, stability, and availability of antibodies suitable for the selective immunoprecipitation of the corresponding protein. We compared the effect of VSV infection on protein synthesis by selective immunoprecipitation of proteins following metabolic incorporation of [^35^S]-methionine from 3–6 hours post infection (Fig 5D). Synthesis of HSP70 3–6 hpi is indistinguishable to that synthesized during a 3h period from mock infected cells (Fig 5D). By contrast, YBX1 synthesis decreases more than two-fold (Fig 5D). This result confirms for 2 cellular mRNAs that the extent of polysome association observed by our RNAseq analysis is reflected in synthesis of the corresponding host proteins.

### Cellular mRNAs exhibit differential polysome distributions following infection

**Fig 6:**
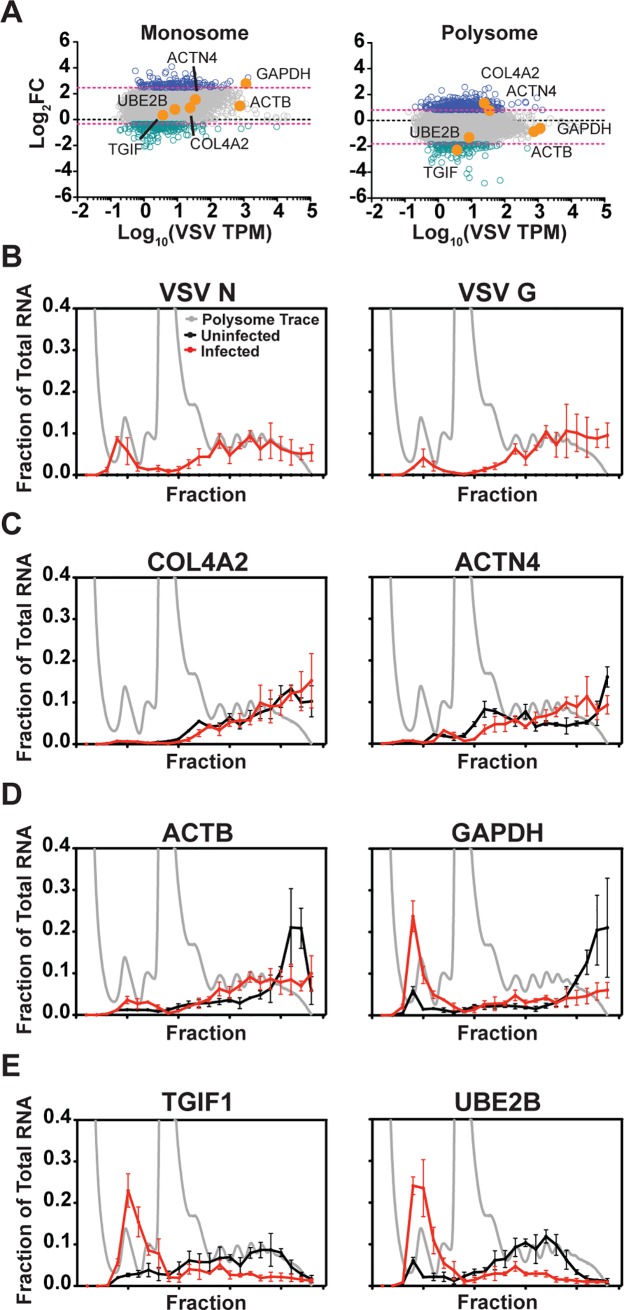
Altered distribution of cellular mRNA within polysomes following infection. (A) The monosome or polysome association of cellular mRNAs at 6 hpi, as determined by RNAseq. Genes picked for validation by polysome profiling and qPCR are highlighted in orange. (B-E) The distribution of mRNA in polysome profiles from uninfected or infected HeLa cells at 6 hpi. A representative polysome trace from infected cells at 6 hpi is shown in light gray, and the mRNA polysome distribution in uninfected cells is shown in black, and infected cells in red. The RNA distribution is presented as the fraction of the total recovered. Error bars denote the standard deviation from three independent replicates. (B) Distribution of VSV N and G mRNA. (C) RNAs with increased polysome association by RNAseq. (D) RNAs with unchanged polysome association following infection. (E) mRNAs with decreased polysome association.

For our experiments we pooled fractions that contained 3 or more ribosomes prior to sequencing of the polysome-associated mRNA. As a result, we do not assess the impact of the redistribution of mRNAs toward smaller polysomes. We therefore selected a subset of cellular mRNAs (Fig 6A), and interrogated their distributions across polysomes using reverse transcription and quantitative PCR. As controls, we analyzed the distribution of N and G mRNAs as representative viral transcripts translated by soluble and endoplasmic reticulum-associated ribosomes, respectively [64]. Consistent with the robust production of viral proteins at 6 hpi, the VSV N and G mRNAs were localized in fractions corresponding to 3 or more ribosomes (Fig 6B). For two cellular mRNAs with increased polysome TPM—collagen type IV alpha 2 (COL4A2) and alpha-actinin-4 mRNA (ACTN4)–the mRNAs remained associated with larger polysomes in infected cells (Fig 6C). Two cellular transcripts that were largely unaltered in their polysome associated TPM–β-actin (ACTB) and glyceraldehyde 3-phosphate dehydrogenase (GAPDH)–remained polysome-associated, although there was a shift toward smaller polysomes and some GAPDH transcripts exited polysomes (Fig 6D). For two representative cellular mRNAs with decreased polysome TPM—transforming growth factor B induced factors (TGIF1) and ubiquitin conjugating enzyme E2 B (UBE2B)–the mRNAs largely exited the polysome fractions, and those that remained were predominantly present on smaller polysomes (Fig 6E). In all cases examined, dissociation of polysomes with EDTA shifted the mRNA distribution toward the fractions corresponding to free ribosomal subunits (S4 Fig). These qPCR data highlight the shift towards smaller polysome fractions for some cellular mRNAs, which also likely contributes to suppression of host protein synthesis. This shift might also explain our finding that HSP70 protein synthesis is relatively unaffected by viral infection (Fig 5D), although the mRNA exhibits increased polysome association.

### Transcripts from viral mutants defective in cap methylation are translated efficiently in infected cells

**Fig 7:**
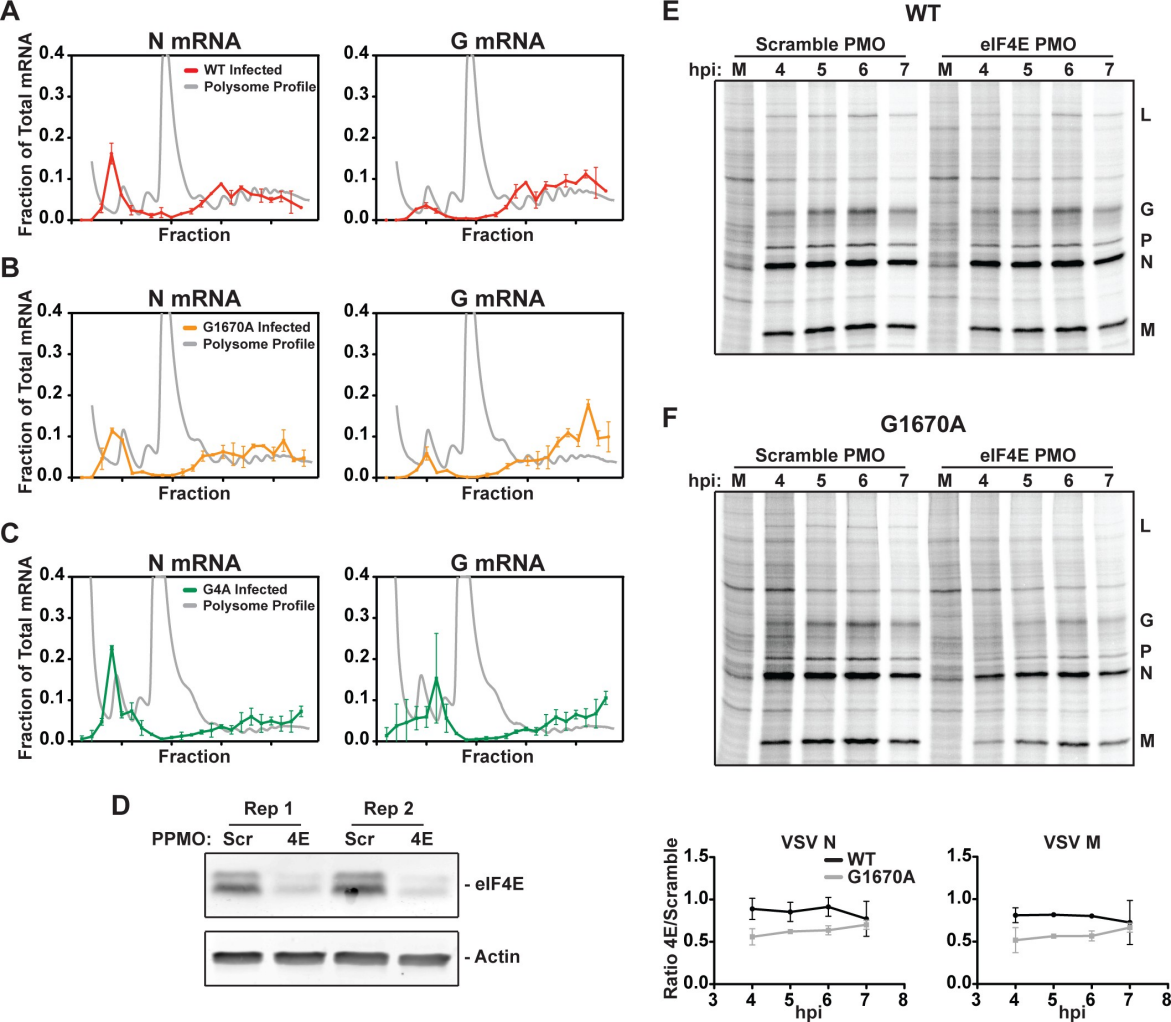
Transcripts from viral mutants defective in cap methylation are translated efficiently in infected cells. (A) Polysome distribution of VSV N and G mRNAs at 6 hpi with wild-type VSV. Results of qPCR for each individual polysome fraction are presented as a fraction of the total recovered, in red. Error bars denote the standard deviation from two independent replicates, and a representative polysome profile from infected cells is shown in gray. (B) Polysome distribution of VSV N and G mRNAs at 6 hpi with a VSV mutant deficient in guanine N-7 cap methylation, L_G1670A_. (C) Polysome distribution of VSV N and G mRNAs at 6 hpi with a VSV mutant, L_G4A_, defective in both guanine-N-7 and ribose-2′-O cap methylation. (D) Western blot showing depletion of eIF4E by PPMO. “Scr” and “4E” denote “scramble” or “eIF4E” PPMO treated cells, respectively. (E-F) Rates of viral protein synthesis in PPMO treated VSV_WT_ or VSV_G1670A_-infected cells following a 10-minute pulse with [^35^S] methionine. A quantitative analysis is presented as a ratio of the rate of synthesis in eIF4E-depleted cells to scramble PMO treated cells. Error bars denote the standard deviation from the mean of two independent replicates.

The abundance of viral mRNA and the suppression of translation initiation through reducing the pool of eIF4E will both contribute to the movement of mRNAs toward smaller polysomes. Recognition of the mRNA cap-structure by eIF4E requires that the guanine-N-7 position of the 7mGpppNmN cap is methylated [65]. We previously reported a panel of recombinant VSVs containing amino acid substitutions within the L-encoded mRNA cap methylase domain that are defective in viral mRNA cap methylation [25]. Mutants VSV-L_G4A_ and VSV-L_G1670A_ contain substitutions in the binding site for the methyl donor s-adenosyl methionine (SAM) and ablate all viral mRNA cap methylation (VSV-L_G4A_) or guanine-N7 but not ribose-2′-O methylation (VSV-L_G1670A_) [25]. As VSV mRNA is relatively insensitive to the loss of eIF4E [44], we would anticipate that the methylation status of the mRNA cap-structure would have little impact on polysome association. Analysis of the distribution of VSV N and G mRNA within polysomes at 6 hpi revealed a similar distribution in cells infected with wild-type virus as well as those infected with VSV-L_G1670A_ and VSV-L_G4A_ (Fig 7A–7C). Correspondingly, the rate of viral protein synthesis in cells infected with VSV-L_WT_ and VSV-L_G1607A_ measured by a 10-minute pulse of [^35^S]-methionine is similar (S5 Fig). These results demonstrate that defects in viral mRNA cap methylation do not significantly alter the rate of viral protein synthesis, consistent with a reduced dependence on eIF4E [25].

To directly test whether manipulating eIF4E levels affects viral translation we depleted eIF4E levels approximately 10-fold using a peptide-conjugated morpholino (PPMO) and measured the rates of VSV-L_WT_ and VSV-L_G1607A_ viral protein synthesis by a 10-minute pulse of [^35^S]-methionine at various times post-infection (Fig 7D–7F). Depletion of eIF4E decreased the rate of viral protein synthesis in VSV-L_1670A_ infected cells, but not L_WT_ infected cells (Fig 7E and 7F). This was not due to sequestration of eIF4E by differential activation of eIF4E-BP1 between VSV-L_WT_ and VSV-L_G1607A_ infected cells, as the kinetics of eIF4E-BP1 dephosphorylation are the same during both infections (S6 Fig). We previously reported that although VSV-L_1670A_ is defective in mRNA cap methylation, up to 20% of the mRNA cap-structures are guanine-N7 methylated. We interpret this finding as indicative of an eIF4E dependent mechanism of translation early in infection.

## Discussion

We obtained two snapshots into the complex battle for control of protein synthesis in cells infected with vesicular stomatitis virus by sequencing of polysome-associated mRNAs at 2 and 6 hpi. Those snapshots provide further evidence that the abundance of vesicular stomatitis virus mRNAs is a determinant of the dominance of viral protein synthesis in infected cells, but highlight several additional attributes of this complex relationship. Those include the demonstration that some host mRNAs that remain polysome associated encode proteins that support viral replication, and some of those that exhibit reduced polysome association encode proteins that are antiviral. We also obtained further insight into the seemingly paradoxical observations that viral infection results in a reduction of the available pool of eIF4E –the rate limiting factor for translation initiation–yet viral mRNAs contain a cap structure that is indistinct to that of host mRNAs. Through the use of a viral mutant defective in mRNA cap methylation, and suppression of eIF4E levels we provide evidence consistent with a transition from an eIF4E dependent phase of viral translation to one less-dependent on eIF4E.

### Evidence in support of a role of mRNA abundance in viral dominance of protein synthesis

The sequence data reported here provides some support for the model that the VSV mRNAs overwhelm the pool of cellular mRNA leading to a redistribution of ribosomes onto viral messages [39]. Evidence in support of this model is based on massively parallel sequencing of mRNAs associated with polysomes, compared with those present in the cytoplasm. As a fraction of the total cytoplasmic mRNA, the VSV mRNAs represent ~1% by 2 hpi, but more than 60% by 6 hpi, illustrating the power of exponential amplification of the viral genome. As a result, the viral N, P, M and G mRNAs far exceed the abundance of any given cellular mRNA, and even the least abundant viral mRNA–that encoding the L polymerase–is present at similar levels to the most abundant cellular mRNA. Thus, one contributor to host cell shut-off in VSV infected cells appears to relate to the synthetic capabilities of the viral polymerase in transcription of viral mRNA.

Similar conclusions have recently been reached for other viruses. Infection of cells with mouse hepatitis virus (MHV) a positive-strand RNA coronavirus that replicates within the cytoplasm results in 80–90% of the cytoplasmic mRNA being viral by 5 hpi [47]. For influenza A virus, a segmented negative-strand RNA virus that replicates in the nucleus, >50% of the total mRNA in the cytoplasm is viral [48]. In this case however, the viral endonuclease PA-X degrades cellular mRNA which further contributes to the dominance of viral mRNA [66, 67]. For vaccinia virus, a DNA virus that replicates entirely within the cytoplasm, degradation of host mRNA through the viral encoded decapping enzymes D9 and D10 also helps the viral mRNAs overwhelm those of the host cell [49, 68, 69]. Collectively these studies indicate that one shared mechanism for host cell shut-off in virus-infected cells is competition for host cell ribosomes through tipping the balance between viral and host mRNA.

Earlier work concludes that viral mRNA abundance is not the determinant of host cell shut-off [41]. When VSV mRNA levels were suppressed up to 14-fold by using defective interfering particles of VSV or a viral mutant defective in transcription, host shut-off was still observed. We did not directly test how suppressing viral mRNA levels impacts host shut-off in this study, but rather conclude that abundance is only part of the mechanism by which the virus induces host cell shut-off–as discussed below.

### A role for eIF4E, the rate limiting factor for translation initiation

We also obtained evidence in support of additional mechanisms that contribute to host cell shut-off in VSV infected cells. We confirmed earlier work that demonstrated a suppression of the pool of the rate limiting factor for initiation, eIF4E, by altering the phosphorylation status of its negative regulator, eIF4E-BP1, which results in eIF4E sequestration [9]. Differences in sensitivity to reductions in eIF4E may contribute to the overrepresentation of cellular mRNA we observe in monosome fractions during infection. We also provide new evidence in support of a phase of VSV gene expression that is dependent on eIF4E through the use of a viral mutant partially defective in guanine-N7 methylation [25] and by the suppression of cellular pools of eIF4E. When eIF4E levels are suppressed 10-fold, we unmask a defect in viral protein synthesis in cells infected with VSV-VSV-L_G1670A_ a mutant with a 4-fold defect in methylation at the guanine-N7-position of the cap-structure. We suspect that this significantly underestimates the eIF4E dependent phase of viral replication since transformed cell lines, like the HeLa cells used here, have higher constitutive levels of eIF4E [70]. Our findings are consistent with a model where viral mRNAs initially compete with cellular mRNAs and translate in an eIF4E dependent manner. As infection progresses and the shut-down of host transcription, mRNA export and eIF4E sequestration continue, the process of initiation is increasingly less dependent on eIF4E. The mechanism by which the viral mRNAs become less dependent on eIF4E remains uncertain, but earlier studies demonstrate that neither the 5′ or 3′ UTR of viral mRNAs facilitate this efficient translation. Ongoing transcription of viral mRNA from the viral genome has also been linked to efficient protein synthesis [46, 71]. Whether this reflects the fact that the virus is an efficient producer of mRNA that supports the competition model, or whether there is a temporal requirement for continued viral mRNA synthesis is uncertain.

### Balancing host and viral gene expression

As obligate intracellular parasites, viruses depend upon host cell functions for their replication. Our sequence analysis provides evidence that VSV infection differentially impacts the polysome association of cellular mRNAs. Several host mRNAs increased in polysome association include genes with known “proviral” functions for entry and replication including heparan sulfate, clathrin, and HSP90 [56, 72–74]. Similarly, host mRNAs with decreased polysome association included genes with published roles in restricting VSV replication such as interferon regulatory factor 1 (IRF1), DDIT4, and TXNIP [62, 63, 75]. It is difficult to definitively determine whether this reflects evolution of the virus to contend with the environment in which it finds itself or a *bona-fide* pro and antiviral effect of a given host protein. Our efforts to address this are confounded by the essential house-keeping nature of many of the proteins encoded by host mRNAs that remain polysome associated. An example of this is provided by enhanced polysome association of eIF3a on VSV infection–a protein that is required for assembly of the multisubunit eIF3 complex. That complex also includes eIF3d which has demonstrated cap-binding ability, and directs eIF4E-independent translation of select mRNAs [76–79]. Depletion of eIF3A, however, resulted in an equivalent reduction in the rates of viral and host translation–inconsistent with a specialized need for eIF3 components in VSV protein synthesis.

### Polysome association and protein synthesis

In this study, we validated that the effect of VSV infection on the polysome association of 2 host mRNAs–HSP70 and YBX1—had a concordant impact on protein synthesis. Although synthesis of HSP70 did not increase per se, this is likely explained by the shifting of many cellular mRNAs towards smaller polysomes. This finding highlights the fact that the designation of RNAs as having “increased” or “decreased” polysome association is imprecise, and reflects the complexities of how any given host gene is regulated. Nevertheless, the general finding that mRNAs with “increased” polysome association on VSV infection are typically larger, have longer half-lives and higher AU content–features that are shared with the viral mRNA–highlights commonalities among mRNAs that remain polysome-associated and thus are more efficient in competing for ribosomes during host shut-off. The cellular mRNAs that exhibit reduced translation efficiency were shorter, have shorter half-lives and higher GC content. Although we validated changes in translatability and differential impacts on viral gene expression for a few cellular genes, it would be of significant interest to perform stable isotope labelling by amino acids in cell culture (SILAC) to non-radioactively label newly synthesized cellular proteins, quantify them on a genome-wide scale and correlate those data with the RNAseq results presented here.

### Integration of different mechanisms of viral suppression of host gene expression

In addition to suppression of host translation through mRNA synthesis and eIF4E manipulation, the VSV matrix protein inhibits host RNA polymerase II transcription [1, 3], and blocks nuclear export of mature mRNAs through complex formation with the nuclear pore components Rae1 and Nup98 [4–8]. A well characterized viral mutant (VSV-M_M51R_) fails to interact with the nuclear pore complex and exhibits a delayed kinetics of host shut-off [6, 10, 80]. A similar analysis to that described here of cells infected with such a mutant may help delineate the extent to which ongoing synthesis and export of cellular mRNA impacts host cell shut-off. We anticipate that over the time course of VSV infection, the contribution of ongoing synthesis and export of host mRNA from the nucleus will result in a relatively modest increase in the fraction of the cytoplasmic mRNA that is cellular.

This study highlights a strategy shared among distinct viruses to commandeer the host translational machinery by outcompeting cellular mRNAs. Precisely where the tipping point between viral and host mRNA levels with respect to this shut-off occurs is uncertain. For VSV, a viral mutant that makes less mRNA than the wild type yet still exhibits host cell shut-off suggests that shut-off can be achieved with less than the 60% of total cytoplasmic mRNA observed here [41]. Additional work will be required to define whether a specific tipping point exists and how this is influenced by other viral strategies such as eIF4E suppression or blocking host gene transcription.

## Materials and methods

### Cells and viruses

HeLa cells (a gift from James Hogle) were maintained in Dulbecco’s modified Eagle medium (DMEM; Invitrogen, Carlsbad, CA) supplemented with 10% fetal bovine serum (FBS; Tissue Culture Biologicals, Tulare, CA). Viral stocks were grown on Syrian golden hamster kidney BSRT7 cells (a gift from K. Conzelmann), and purified on linear 15–45% sucrose gradients prepared in NTE (10 mM Tris pH 7.4, 100 mM NaCl, 1 mM EDTA). Viral titers were determined by plaque assay on African green monkey kidney Vero cells (ATCC, CCL-81). For infections, cells were first washed with Hanks’ Balanced Salt Solution (HBSS) and incubated with virus for 1 hour at 37°C in serum free medium, washed with HBSS and subsequently incubated with medium supplemented with 2% FBS.

### Polysome profiling

For polysome profiling, HeLa cells were treated with DMEM containing 100 μg ml^-1^ cycloheximide at 37°C for 3 minutes. Cells were washed twice with 1X ice-cold phosphate buffered saline (PBS) containing 100 μg ml^-1^ cycloheximide, and kept on ice or at 4°C. Cells were scraped into a 1.5 ml microcentrifuge tube in 1X PBS with 100 μg ml^-1^ cycloheximide, and pelleted at 300 ✕ *g* for 10 minutes. Cells were resuspended in 250 μl of a hypotonic buffer of 5 mM Tris (pH 7.4), 2.5 mM MgCl_2_, 1.5 mM KCl, and RNAsin (Promega, Madison, WI), supplemented with cycloheximide to 100 μg ml^-1^ and DL-Dithiothreitol (DTT) to 3 μM. The detergents Triton X-100 0.5% (vol/vol) and sodium deoxycholate 0.5% (wt/vol) were then added sequentially, cells were briefly vortexed, and incubated for 15 minutes on ice and clarified by centrifugation at 12,000 ✕ *g* for 2 min. Polysomes were separated on sucrose gradients prepared on a Gradient Master Station (Biocomp, Fredericton, Canada) using 10% and 50% (wt/vol) sucrose dissolved in 15 mM Tris (pH 7.4), 15 mM MgCl_2_, and 150 mM NaCl supplemented with RNAsin and 100 μg ml^-1^ cycloheximide. Following centrifugation for 2 hours at 40,000 ✕ *g* in a Beckman Coulter ultracentrifuge using an SW40Ti rotor, 500 μl fractions were collected from the top of the gradient while monitoring absorbance at λ = 254 nm on a Gradient Master Station.

### RNA extraction, library preparation, and RNAseq

RNA was extracted from total cytoplasmic, polysome, or monosome fractions using LS Trizol (Invitrogen) according to the manufacturer’s protocol. Equal amounts of RNA as determined by spectrophotometry using absorbance at 260 nm on a Nanodrop 2000 (Thermo Fisher, Waltham, MA) were subject to library preparation using the Illumina TruSeq vII RNA Library Preparation Kit (Illumina, San Diego, CA), and sequenced at the Whitehead Institute (Cambridge, MA) on an Illumina HiSeq2500. Reads were trimmed and mapped to the concatenated hg38 and VSV genomes using CLC Genomics Workbench (Qiagen, Redwood City, CA). Mapping parameters were as follows; mismatch cost 2, insertion cost 3, deletion cost 3, length fraction 0.8, similarity fraction 0.8, and a maximum of 10 hits per read. Raw sequence data is available from the NCBI Sequence Read Archive (SRA) under the primary accession code SRP158625.

### Gene expression analysis

Transcripts per Kilobase Million (TPM) was calculated for genes with 56 or more mapped reads in the cytoplasmic fraction of both uninfected and infected cells using the total number of mapped exon reads. To identify cellular mRNAs that were potential targets for translational regulation in infected cells, we determined the TPM in the polysome fraction/TPM in the total cytoplasmic fraction for each individual mRNA. This ratio was determined for uninfected and infected cells, and presented as the log_2_ fold change. Gene ontology analysis was performed in R using GOseq.

### mRNA characteristics and statistical analysis

UTRs and CDS sequences were downloaded from the UCSC table browser using “KnownCanonical” mRNA identifiers. Non-protein coding RNAs were excluded from the analysis. Poly (A) tail length and mRNA half-lives were from published data sets [54, 55]. Graphs and statistical analyses were performed in R using the “wilcox_test” statistical test, the “density” kernel density estimation, and “geom_boxplot” or “geom_density” functions in ggplot and cowplot.

### RT-qPCR

Total RNA was recovered from polysome fractions using LS Trizol according to the manufacturer’s protocol. RNA (500 ng) was annealed with oligo d(T)_20_ and reverse-transcribed using Superscript III Reverse Transcriptase (Thermo Fisher) at 50°C for 1 hour. Following digestion of the RNA strand with RNaseA and RNaseH for 15 min at 37°C, reactions were diluted 1:5 for cellular gene-specific qPCR or 1:125 for viral gene-specific qPCR. Quantitative PCR was performed using Power Sybr Green (Thermo Fisher) and relative amounts determined by ΔΔC_t_. Forward (F) and Reverse (R) primers were as follows:

ACTB (F) 5′ ACCCAGCACAATGAAGATCA 3′, (R) 5′ CTCGTCATACTCCTGCTTGC 3′;

ACTN4 (F) 5′ ACATCTCCGTGGAAGAGACC 3′, (R) 5′ GGAAGTTCTGCACATTGACG 3′;

COL4A2 (F) 5′ AACGGGATTCCATCAGACAC 3′, (R) 5′ ATGCCTCTTATTCCTGGTTCC 3′;

DDIT4 (F) 5′ CGGAGGAAGACACGGCTTA 3′, (R) 5′ ACAAGTGTTCATCCTCAGGGT 3′;

GAPDH (F) 5′ AGCCTCAAGATCATCAGCAATG 3′, (R) 5′ ATGGACTGTGGTCATGAGTCCTT 3′;

TGIF1 (F) 5′ CACCGTTACAATGCCTATCC 3′, (R) 5′ GATTTGGATCTTTGCCATCC 3′;

UBE2B (F) 5′ CAATTCAGTCTCTGCTGGATG 3′, (R) 5′ AACAATGGCCGAAACTCTTT 3′;

VSV G (F) 5′ GTGGGATGACTGGGCTCCAT 3′, (R) 5′ CTGCGAAGCAGCGTCTTGAA 3′;

VSV N (F) 5′ GAGTGGGCAGAACACAAATG 3′, (R) 5′ CTTCTGGCACAAGAGGTTCA 3′

Polysome distribution is presented as the fraction of total recovered RNA for each individual polysome fraction.

### Chemical, siRNA or morpholino inhibition of host factors

For inhibition of HSP90, cells were incubated throughout the course of the experiment in 2.5 μM 17-[2-(Dimethylamino)ethyl]amino-17-desmethoxygeldanamycin (17-DMAG; AdipoGen Corp, San Diego, CA). For siRNA depletions, cells were pretreated with siRNAs against DDIT4 (D-010855-01), eIF3a (D-019534-03), or non-targeting siRNA #2 (D-001210-02), and #3 (D-001210-03) Dharmacon (Lafayette, CO) for 48 h. Briefly, Lipofectamine 2000 (Thermo Fisher) was diluted 100-fold in Optimem (Invitrogen), and incubated for 15-minutes with an equal volume of siRNA in Optimem. Reverse transfections were performed in 24 well plates with 5.5 x 10^4^ HeLa cells per well at a final concentration of 50 nM siRNA in a total volume of 600 μl.

Images of rVSV-eGFP infected cells were acquired using a 10× objective on a Nikon Eclipse TE300 microscope (Nikon Instruments, Melville, NY) equipped with a Spot RT SE18 Monochrome camera (Diagnostic Instruments, Sterling Heights, MI). For cytometry, cells were washed in HBSS, trypsinized, fixed in 4% paraformaldehyde at 4°C for 15 minutes and measured using a FACSCalibur (Cytek Development, Freemont, CA). Cytometry data was analyzed using FlowJo (FlowJo Inc, Ashland, OR). For mean fluorescence intensity (MFI) we gated on live cells identified by forward and side-scatter. To measure the % infected cells we subtracted those cells that fell within the gate established from uninfected control cells.

For luciferase assays, where indicated HeLa cells were transfected with siRNA, and the medium replenished at 24 h. Cells were transfected 6 h later with 25 ng pRL-CMV (Promega), and activity measured 24 h later. For viral driven luciferase reporters siRNA transfected cells were infected at 48 h and monitored 6 h later. Luciferase expression was measured in a SpectraMax L microplate reader using the appropriate reagents according to the manufacturer’s instructions (Promega, E1501 and E2810).

For depletion of host factors by peptide-conjugated morpholinos (PPMOs) approximately 2.0 x 10^4^ HeLa cells per well of a 24 well plate were treated 24 h later with 15 μM of the indicated PPMO. At 24 h post treatment, the media was replaced with fresh medium containing 15 μM PPMO, and used for testing 24 h later.

### Western blots

Cells were washed twice with ice-cold 1X PBS and lysed in Rose Lysis Buffer consisting of 10 mM Tris-HCl (pH 7.4), 66 mM EDTA, 0.4% w/v sodium deoxycholate, and 1% v/v NP-40 on ice for 15 minutes. Rose Lysis Buffer was supplemented with Phosphatase Inhibitor Cocktail 2 (Sigma-Aldrich, St. Louis, MO) and Halt Protease and Phosphatase Inhibitor Cocktail (Thermo Fisher) for detection of phospho-eIF4E-BP1. Lysates were clarified, protein input was normalized by Bradford Assay and proteins resolved on polyacrylamide gels– 10% for eIF3a and eIF4E or 12%, eIF4E-BP1. Proteins were transferred to nitrocellulose membranes for 90 minutes, eIF4E and eIF4E-BP1, or 120 minutes, eIF3a, at 100v. Membranes were blocked with Odyssey Blocking Buffer in PBS (LI-COR, Lincoln, NE) for 1 h at room temperature, and incubated overnight at 4°C with the following primary antibodies: rabbit anti-eIF3a (Cell Signaling, #3411), rabbit anti-eIF4E (Cell Signaling, #9742), rabbit anti-4E-BP1 (Cell Signaling, #9452), rabbit anti-phospho-4E-BP1 Ser65 (Cell Signaling, #9451), rabbit anti-phospho-4E-BP1 Thr37/46 (Cell Signaling, #2855), mouse anti-actin (Millipore, #MAB1501), mouse anti-actin (Sigma, #A5316). Membranes were washed 3X with 1X PBS-T for 5 minutes at room temperature, and incubated with the relevant secondary antibodies: goat anti-mouse IRDye 680RD (LI-COR, #925–68070) or goat anti-rabbit IRDye 800CW (LI-COR, #925–32211), for 1 hour at room temperature. Membranes were washed again and kept in 1X PBS, and scanned on an Odyssey CLx (LI-COR).

### Metabolic labeling of proteins and immunoprecipitation

HeLa cells were starved in DMEM lacking L-methionine (Corning, #17-204-Cl) for 30 minutes, prior to addition of [^35^S] Express Protein Labeling Mix (Perkin Elmer, Waltham, MA) at 0.11 mCi ml^-1^. Cell lysates were prepared as described above and separated on a low-bis 10% polyacrylamide gel. The gel was dried for 1.5 h at 80°C in a vacuum gel drier and detected using a phosphoimager. Quantitative analyses of band intensities was performed in ImageQuant TL v8.1 (GE Healthcare, Marlborough, MA).

For radioimmunoprecipitations, 4.0 x 10^6^ HeLa cells plated in 10 cm dishes (Corning) were starved of methionine for 1 h at 24 h post-plating, and labeled with [^35^S]-Express for 3 h. Cells were washed twice with ice-cold 1X PBS, collected by scraping and subsequent centrifugation for 2 minutes at 4°C and 2,000 ✕ *g* and lysed in 1 ml of 50 mM Tris (pH 7.4), 150 mM NaCl_2_, 1 mM EDTA, 1% v/v NP40, 2 mM DTT, supplemented with Pierce Protease Inhibitor (Thermo Fisher) on ice for 15 minutes. Protein input was normalized by Bradford Assay, SDS was added to 0.1%, and 450 μl lysate was pre-cleared for 1h at 4°C on a nutator with 50 μl pre-washed Pierce Protein A Agarose Beads. Protein A beads were pelleted by centrifugation for 2 minutes at 4°C and 2,000 ✕ *g* and the labeled supernatant incubated with primary antibody at 4°C overnight. The antibodies used for immunoprecipitation were 4 μg anti-YB1 (Abcam, #ab76149) and 5 μg anti-HSP70 (Enzo, #ADI-SPA-822). Immune complexes were isolated using 50 μl pre-washed Pierce Protein A Agarose Beads, by incubating for 4 hours at 4°C. Bead complexes were collected by centrifugation for 2 minutes at 4°C and 2,000 ✕ *g*, washed 5X with 950 μl ice-cold IP lysis buffer with 0.1% SDS, on an orbital shaker for 3 minutes at room temperature. Protein-antibody complexes were eluted by boiling in 4X SDS loading buffer, the beads pelleted for 2 minutes at 4°C in a microcentrifuge and the supernatant loaded on a 10% polyacrylamide gel. After electrophoresis the gel was dried and imaged using a phosphoimager.

### Metabolic labeling of viral RNA

Approximately 1.5 x 10^6^ HeLa cells were plated per well of a 6-well dish and 24 hours later incubated with phosphate free media (Gibco, 11971–025) for 1 h. Thirty minutes before infection, actinomycin D-mannitol (Sigma, #A5156) was added to a final concentration of 5 μg ml^-1^. Infections were carried out in phosphate free media supplemented with 10 μg ml^-1^ actinomycin D and 2.5 μM 17-DMAG. At 1 hpi cells were washed, fresh medium added, and supplemented 3 h later with [^32^P]-orthophosphoric acid (Perkin Elmer, #NEX053H) 0.20 μCi μl^-1^. Cells were harvested at 6 hpi in Rose Lysis Buffer, and total RNA was extracted using LS Trizol. RNA was separated on a 6M Urea-Agarose gel as previously described, and detected using a phosphoimager [81].

## Supporting information

S1 Fig The viral gene cascade at 2 and 6 hpi. (A) Sequence read coverage of the viral genome in the cytoplasmic or polysome fractions at 6 hpi. Analysis was performed in CLC Genomics Workbench. (B) Distribution of viral mRNAs present in monosome and polysome fractions at 2 hpi mapped to the viral genome. Expression level is presented as Transcripts per Kilobase Million (TPM) to normalize for gene length and library size, error bars denote standard deviation from two biological replicates. (C) Viral mRNA distribution at 6 hpi, presented as in B.
(TIF)

S2 Fig High AU content is a general feature of cellular mRNAs that remain most polysome-associated on infection. (A-F) Analysis of mRNA characteristics for RNAs with increased (blue) or decreased (green) polysome association as compared to mRNAs with polysome association measurements at 6 hpi within 2 standard deviations of the mean (gray). ***p<2.2 x 10^−16^, **p<5.05 x 10^−5^, *p<0.05; all other p>0.05 as determined by the Wilcoxon rank sum test compared to mRNAs within the 95% confidence interval. Hinges correspond to the 25^th^-75^th^ percentiles, and whiskers denote 1.5 times the inter-quartile range. CDS is defined as the coding region of the gene sequence.
(TIF)

S3 Fig Effect of perturbing host gene function on viral replication. (A) Brightfield and eGFP images of rVSV-eGFP infected HeLa cells treated with 17-DMAG, refers to Fig 5. Below, a representative histogram of flow data, and quantitative analysis of GFP positive cells by flow cytometry, normalized to vehicle. Error bars denote standard deviation from the mean of three independent replicates. To the right is viral RNA transcription from 3–6 hpi in actinomycin D and 17-DMAG treated HeLa cells. The position of viral RNAs is noted on the right. Shown is a representative gel from three independent replicates. (B) Top panel, western blot for eIF3a protein 48 hours post-siRNA transfection. Presented is a representative western blot from three independent replicates. The bottom two panels show the brightfield images for Fig 5 and percent infected cells, as in A. (C) Top panel showing the knockdown efficiency of DDIT4 RNA by RT-qPCR 48 hours post siRNA transfection. The bottom two panels show cell density and rVSV-eGFP expression, presented as in A except that GFP quantification was normalized to a non-targeting control siRNA.
(TIF)

S4 Fig Messenger RNAs are ribosome-associated at 6 hpi. (A-E) The distribution of mRNA in polysome profiles from uninfected or infected HeLa cells at 6 hpi in the presence or absence of EDTA. EDTA was added to a final concentration of 50 mM and lysates were incubated for 5 minutes on ice. Lysates were sedimented through gradients containing EDTA. Cycloheximide was omitted from all experimental conditions, including non-EDTA treated lysates. A representative polysome trace from infected cells at 6 hpi following EDTA treatment is shown in light gray. The mRNA polysome distribution from untreated, uninfected lysates is shown in black, and untreated, infected cells in red. EDTA treated uninfected distributions are shown in dashed gray, and EDTA treated infected cell lysates in dashed pink. The RNA distribution is presented as the fraction of the total amount of a given RNA recovered. A representative experiment from two independent replicates is presented. Polysome distributions of (A) VSV N, (B) VSV G, (C) ACTN4, (D) ACTB, and (E) UBE2B.
(TIF)

S5 Fig Efficient protein synthesis by a viral mutant defective in mRNA cap methylation. (A) Phosphoimage analysis of an SDS-PAGE of proteins synthesized in cells following a 10-minute pulse with [^35^S]-methionine at the indicated times post-infection. Cells were infected with VSV_WT_ or a mutant defective in guanine-N7 cap-methylation, VSV_G1670A_. The position of viral proteins is shown to the right, and the gel presented is a representative gel of three independent replicates.
(TIF)

S6 Fig The kinetics of eIF4E-BP1 activation is the same between VSV_WT_ and VSV_G1670_ infected cells. (A) Western blot using an antibody that detects total eIF4E-BP1. Infected cells were lysed at the indicated times post-infection in the presence of phosphatase inhibitors, and the lysates separated on 12% polyacrylamide gels. The same samples were used for panels A-C. Actin is used to show equal loading. Tor denotes samples treated with torin, a positive control for eIF4E-BP1 dephosphorylation. (B) Western blot for Serine 65 phosphorylated eIF4E-BP1. (C) Western blot for Threonine 37/46 phosphorylated eIF4E-BP1. The blots presented are representative of two independent replicates.
(TIF)

S1 Dataset 2 hpi normalized gene expression results. (TXT)

S2 Dataset 6 hpi normalized gene expression results. (TXT)

S3 Dataset Results of GO analysis. (XLSX)
