# Supplementary figures and images for: Global analysis of polysome-associated mRNA in vesicular stomatitis virus infected cells
Source: PLoS Pathog. 2019 Jun 21;15(6):e1007875. doi: 10.1371/journal.ppat.1007875 (PMC6608984; doi:10.1371/journal.ppat.1007875)

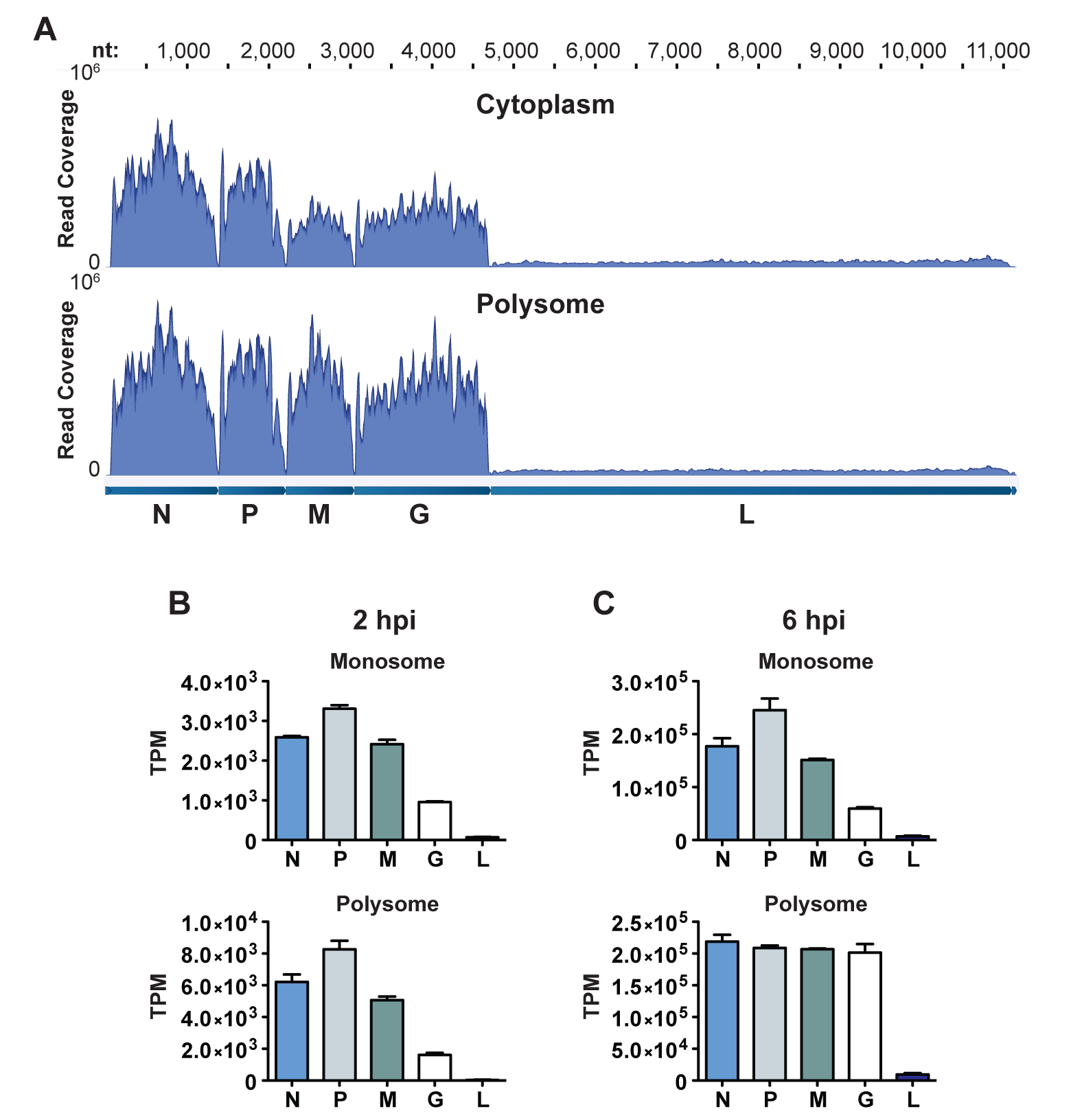

Supplement: S1 Fig — (A) Sequence read coverage of the viral genome in the cytoplasmic or polysome fractions at 6 hpi. Analysis was performed in CLC Genomics Workbench. (B) Distribution of viral mRNAs present in monosome and polysome fractions at 2 hpi mapped to the viral genome. Expression level is presented as Transcripts per Kilobase Million (TPM) to normalize for gene length and library size, error bars denote standard deviation from two biological replicates. (C) Viral mRNA distribution at 6 hpi, presented as in B. (TIF) [file ppat.1007875.s001.tif]

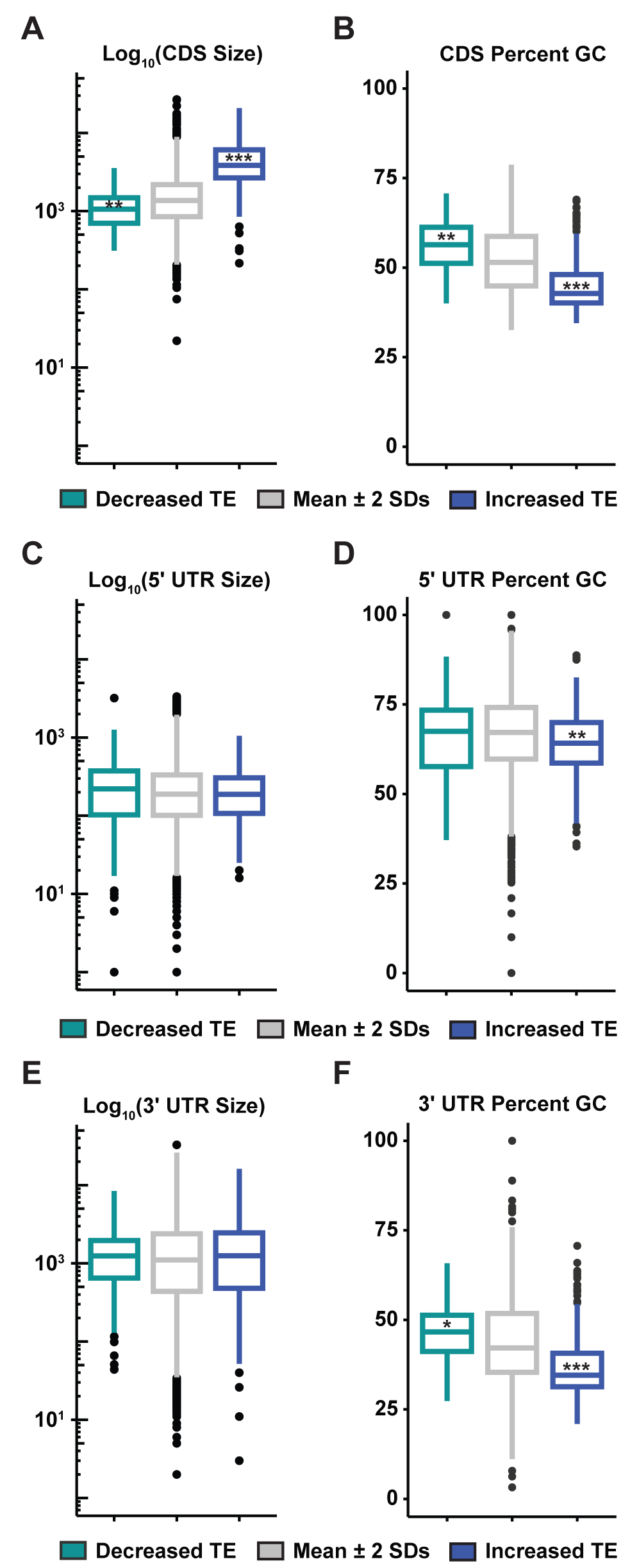

Supplement: S2 Fig — (A-F) Analysis of mRNA characteristics for RNAs with increased (blue) or decreased (green) polysome association as compared to mRNAs with polysome association measurements at 6 hpi within 2 standard deviations of the mean (gray). ***p<2.2 x 10^−16^, **p<5.05 x 10^−5^, *p<0.05; all other p>0.05 as determined by the Wilcoxon rank sum test compared to mRNAs within the 95% confidence interval. Hinges correspond to the 25th-75th percentiles, and whiskers denote 1.5 times the inter-quartile range. CDS is defined as the coding region of the gene sequence. (TIF) [file ppat.1007875.s002.tif]

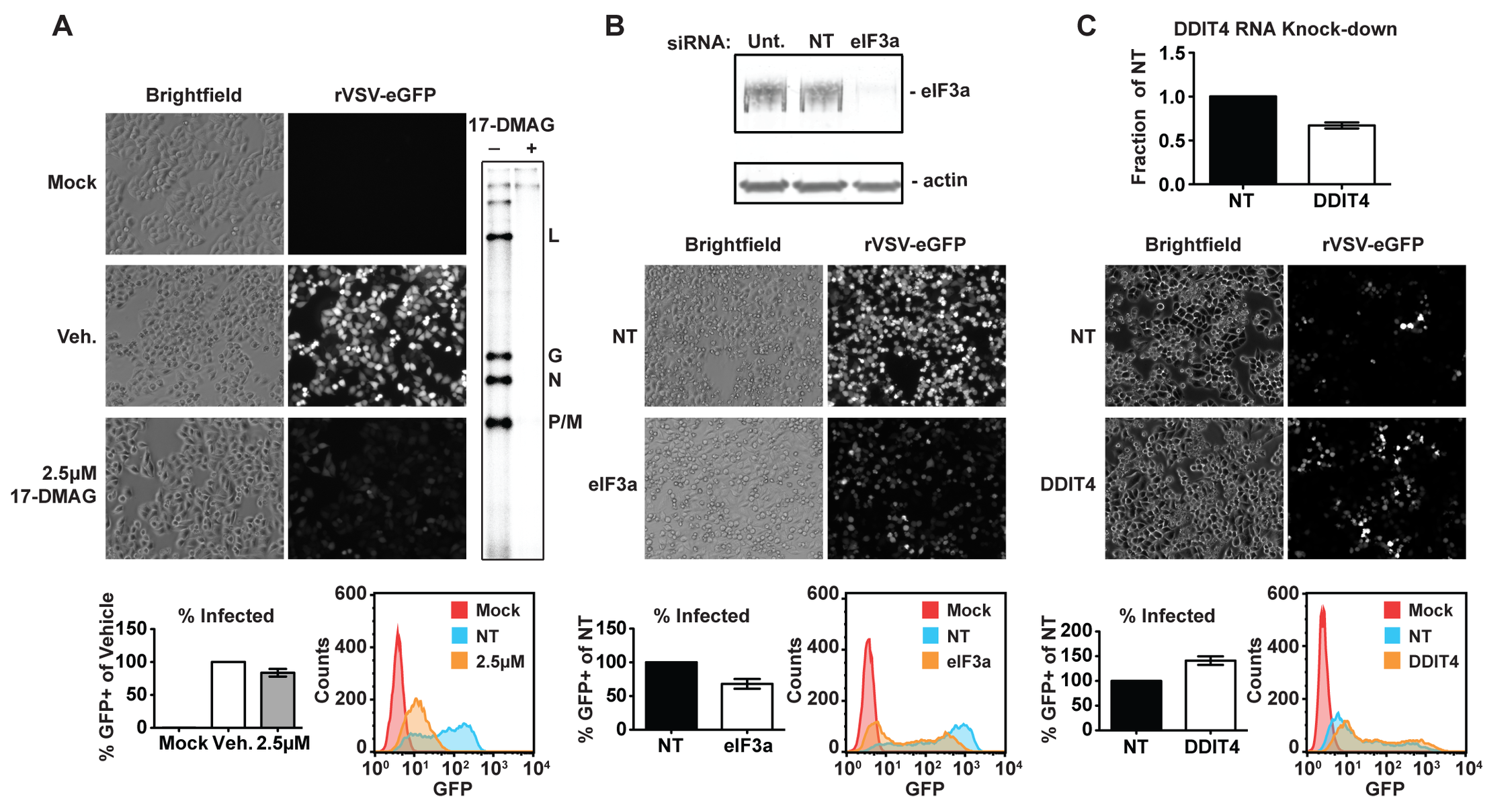

Supplement: S3 Fig — (A) Brightfield and eGFP images of rVSV-eGFP infected HeLa cells treated with 17-DMAG, refers to Fig 5. Below, a representative histogram of flow data, and quantitative analysis of GFP positive cells by flow cytometry, normalized to vehicle. Error bars denote standard deviation from the mean of three independent replicates. To the right is viral RNA transcription from 3–6 hpi in actinomycin D and 17-DMAG treated HeLa cells. The position of viral RNAs is noted on the right. Shown is a representative gel from three independent replicates. (B) Top panel, western blot for eIF3a protein 48 hours post-siRNA transfection. Presented is a representative western blot from three independent replicates. The bottom two panels show the brightfield images for Fig 5 and percent infected cells, as in A. (C) Top panel showing the knockdown efficiency of DDIT4 RNA by RT-qPCR 48 hours post siRNA transfection. The bottom two panels show cell density and rVSV-eGFP expression, presented as in A except that GFP quantification was normalized to a non-targeting control siRNA. (TIF) [file ppat.1007875.s003.tif]

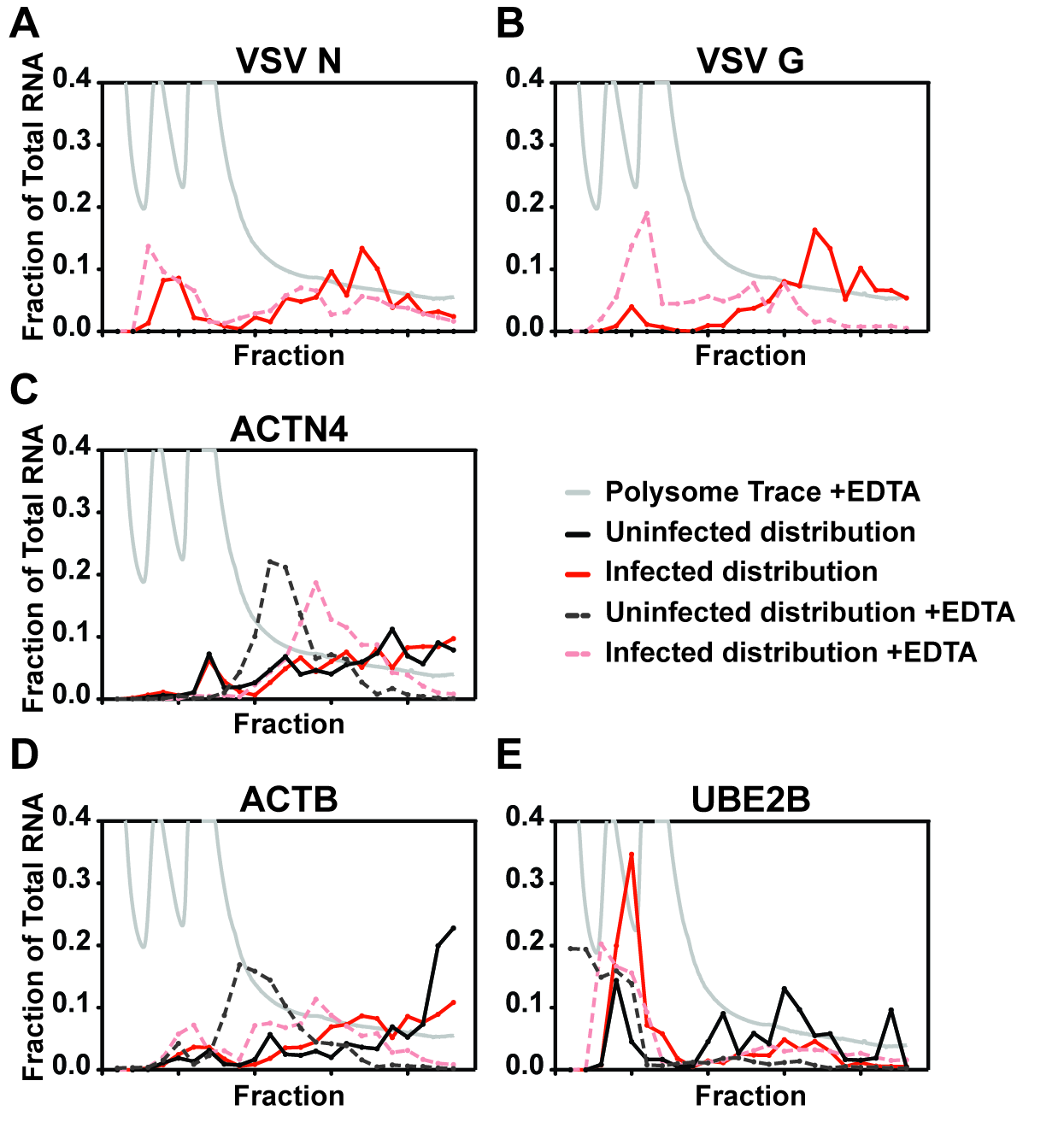

Supplement: S4 Fig — (A-E) The distribution of mRNA in polysome profiles from uninfected or infected HeLa cells at 6 hpi in the presence or absence of EDTA. EDTA was added to a final concentration of 50 mM and lysates were incubated for 5 minutes on ice. Lysates were sedimented through gradients containing EDTA. Cycloheximide was omitted from all experimental conditions, including non-EDTA treated lysates. A representative polysome trace from infected cells at 6 hpi following EDTA treatment is shown in light gray. The mRNA polysome distribution from untreated, uninfected lysates is shown in black, and untreated, infected cells in red. EDTA treated uninfected distributions are shown in dashed gray, and EDTA treated infected cell lysates in dashed pink. The RNA distribution is presented as the fraction of the total amount of a given RNA recovered. A representative experiment from two independent replicates is presented. Polysome distributions of (A) VSV N, (B) VSV G, (C) ACTN4, (D) ACTB, and (E) UBE2B. (TIF) [file ppat.1007875.s004.tif]

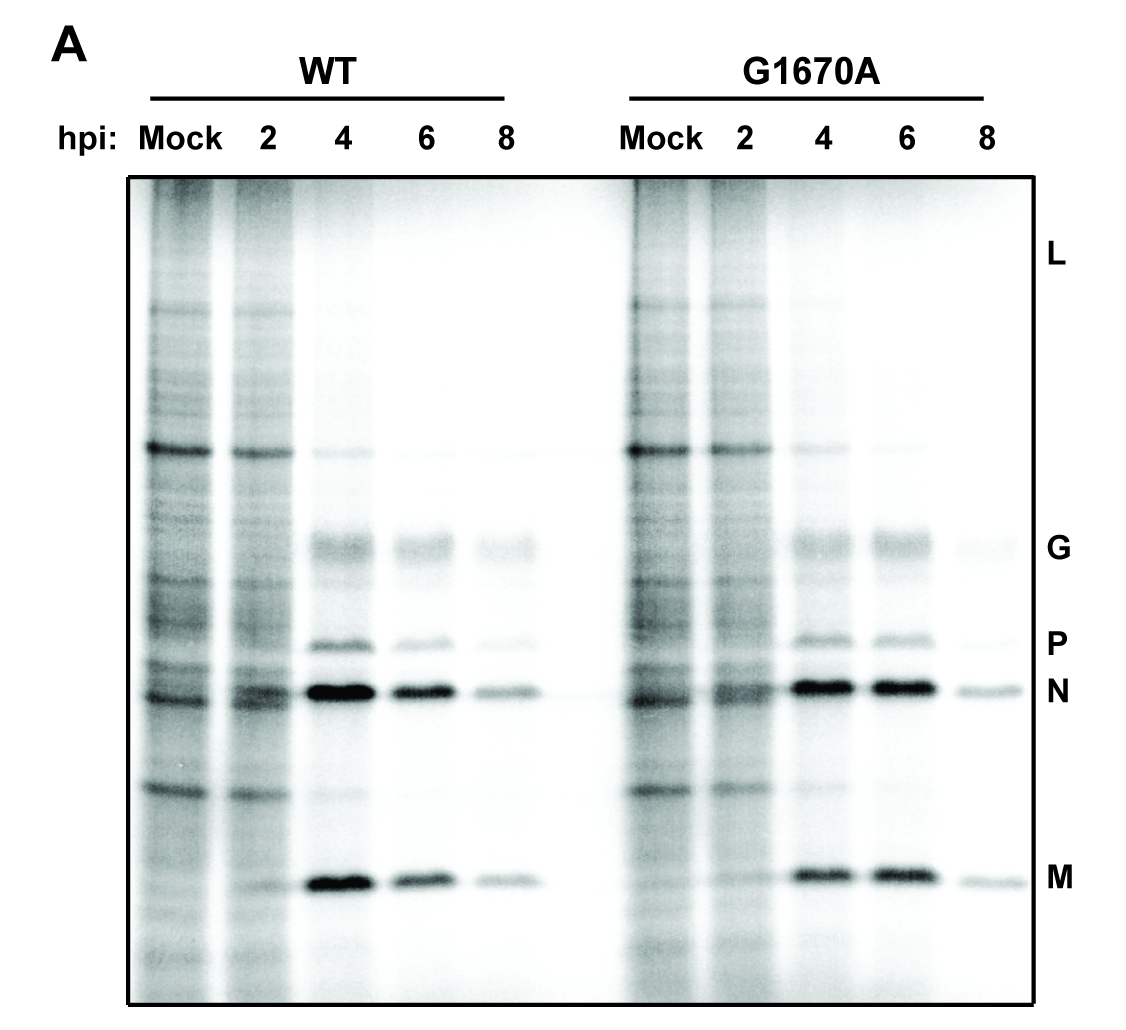

Supplement: S5 Fig — (A) Phosphoimage analysis of an SDS-PAGE of proteins synthesized in cells following a 10-minute pulse with [35S]-methionine at the indicated times post-infection. Cells were infected with VSVWT or a mutant defective in guanine-N7 cap-methylation, VSVG1670A. The position of viral proteins is shown to the right, and the gel presented is a representative gel of three independent replicates. (TIF) [file ppat.1007875.s005.tif]

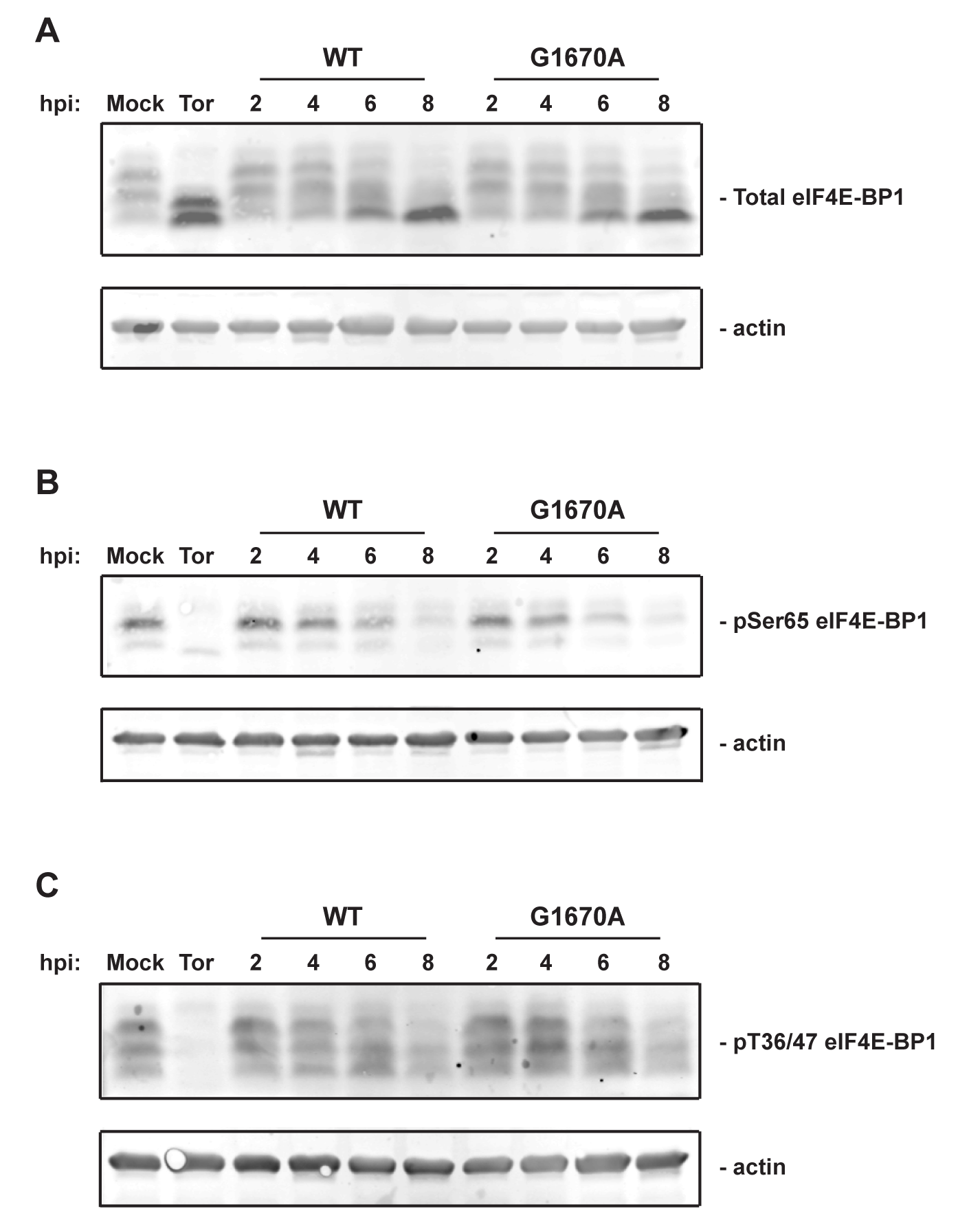

Supplement: S6 Fig — (A) Western blot using an antibody that detects total eIF4E-BP1. Infected cells were lysed at the indicated times post-infection in the presence of phosphatase inhibitors, and the lysates separated on 12% polyacrylamide gels. The same samples were used for panels A-C. Actin is used to show equal loading. Tor denotes samples treated with torin, a positive control for eIF4E-BP1 dephosphorylation. (B) Western blot for Serine 65 phosphorylated eIF4E-BP1. (C) Western blot for Threonine 37/46 phosphorylated eIF4E-BP1. The blots presented are representative of two independent replicates. (TIF) [file ppat.1007875.s006.tif]
